# Evaluation of Health Associations With Height‐Normalised Abdominal Body Composition Indices: A Single‐Centre Cross‐Sectional Study

**DOI:** 10.1002/jcsm.13609

**Published:** 2024-10-07

**Authors:** Yupeng Liu, Hangqian He, Keyu Qian, Yufeng Huang, Xuemei Ao, Xudong Shi, Binye Ruan, Ru Xue, Xiaoyi Fu, Shuran Wang

**Affiliations:** ^1^ Department of Preventive Medicine, School of Public Health and Management Wenzhou Medical University Wenzhou China

**Keywords:** adipose tissue, allometric analysis, health outcomes, height, scaling powers

## Abstract

**Background:**

Traditional metrics such as body mass index (BMI) and waist circumference (WC) fail to accurately assess the health outcomes associated with abdominal adiposity, because they neglect the intricacies of adipose tissue distribution. Notably, the variability in body composition scaled to height remains underexplored in Chinese demographics. This study introduces height‐normalised indices of abdominal adiposity using computed tomography (CT) scans and further assesses their associations with various health outcomes.

**Methods:**

In a large, diverse Chinese population (*n* = 1054 healthy individuals; *n* = 1159 with dyslipidemia; *n* = 803 with diabetes; *n* = 1289 with cardio‐cerebrovascular diseases; *n* = 1108 with cancers; and *n* = 509 with abnormal bone mas), abdominal CT scans were performed and allometric growth model analyses were used to derive height‐normalised indices (body composition/height^*β*^). Logistic regression models assessed the associations between these indices and health outcomes.

**Results:**

Distinct scaling powers were observed for visceral adipose tissue (VAT), subcutaneous adipose tissue (SAT) and total abdominal adipose tissue (TAT), as well as for sagittal diameter (SAD), with marked sex differences. Powers for VAT were 1.786 ± 1.270 for males and 1.274 ± 0.692 for females. Powers for SAT were 2.266 ± 0.856 for males and 1.656 ± 0.497 for females. Powers for TAT were 2.141 ± 0.967 for males and 1.438 ± 0.489 for females. Powers for SAD were 0.646 ± 0.217 for males and 0.678 ± 0.141 for females. After controlling for age, BMI and WC, VAT/height^*β*^, TAT/height^*β*^ and SAD/height^*β*^ retained their significantly positive associations with the odds of health outcomes, whereas SAT/height^*β*^ did not.

**Conclusions:**

Our findings endorse the clinical utility of height‐normalised indices, particularly VAT/height^*β*^, TAT/height^*β*^ and SAD/height^*β*^, in health outcomes assessment. These indices, grounded in robust empirical data, underscore the necessity of a nuanced approach in obesity‐related health evaluations, advocating for a departure from conventional methods like BMI.

## 1 Introduction

Obesity assessment has conventionally hinged on the body mass index (BMI) [1]—a rudimentary metric that, while easy to calculate, often oversimplifies the multifaceted nature of adipose tissues and their health implications. Specifically, BMI's broad‐stroke approach fails to differentiate between visceral adipose tissue (VAT) and subcutaneous adipose tissue (SAT), a critical distinction in understanding obesity‐related health risks [2].

Recent insights reveal that abdominal adiposity, particularly VAT, correlates strongly with metabolic anomalies, such as insulin resistance, and conditions including diabetes, hypertension, dyslipidemia and certain cancers [3, 4, 5, 6, 7]. These associations underscore the need for a more nuanced metric that captures the complexities of adipose tissue distribution. Current research often employs direct measurements of abdominal fat areas through CT or MRI [8, 9, 10, 11, 12], offering detailed insights yet overlooking the potential influence of an individual's height.

Our research addresses this significant gap, pioneering an investigation into how abdominal adipose tissue scales with height, particularly within a substantial and diverse Chinese cohort. By proposing novel height‐normalised indices (body composition/height^*β*^), our study transcends traditional limitations, offering a refined lens through which to view the obesity‐health paradigm. Our aim extends beyond mere measurement, seeking to equip healthcare professionals with a more sophisticated understanding that could revolutionise obesity management strategies. This innovative approach, we believe, will serve as a catalyst for more personalised, effective public health interventions globally.

## 2 Methods

### 2.1 Subjects

In this study, we initially reviewed the records of 5362 individuals who had undergone abdominal CT scans between September 2020 and May 2022 at the Second Affiliated Hospital of Wenzhou Medical University.

After this initial screening, we identified a healthy population of 1054 individuals. Additionally, we recognised 1159 subjects diagnosed with dyslipidemia, determined by established criteria (triglycerides ≥ 2.26 mmol/L or total cholesterol ≥ 6.22 mmol/L or low‐density lipoprotein cholesterol ≥ 4.14 mmol/L or high‐density lipoprotein cholesterol < 1.04 mmol/L). Our study also included 803 subjects diagnosed with type 2 diabetes, as determined by standard diagnostic thresholds (fasting plasma glucose ≥ 7.0 mmol/L, 2‐h plasma glucose after a 75‐g oral glucose tolerance test ≥ 11.1 mmol/L, glycated haemoglobin ≥ 6.5% or random plasma glucose ≥ 11.1 mmol/L with hyperglycemia symptoms). Furthermore, we included 1289 patients diagnosed with cardio‐cerebrovascular diseases and 1108 patients diagnosed with cancers based on discharge records. We used *T*‐score and *Z*‐score to express bone mineral density. Following WHO criteria, we classified individuals as having normal bone density (*T*‐score ≥ −1.0 or *Z*‐score > −2.0), osteopenia (−2.5 < T‐score < −1.0 or *Z*‐score ≤ − 2.0) or osteoporosis (T‐score ≤ − 2.5). We considered both osteopenia and osteoporosis as indicators of abnormal bone mass, which included 509 individuals in our study. The flow diagram of participants is presented in Figure S1.

### 2.2 Measurements of Body Composition

We used computed tomography (CT), which is the gold standard technique and provides rapid imaging capability and high‐resolution output [13], to accurately assess body composition. Body composition measurements included VAT area, SAT area, waist circumference, transverse diameter and sagittal diameter. Participants were positioned supine with both arms stretched above their heads. A Philips Brilliance 16‐slice CT machine was used to perform a scan at the level of the third lumbar vertebra (L3). Cross‐sectional images of abdominal adipose tissue were manually identified and extracted in the Digital Imaging and Communications in Medicine format. ImageJ software (version 1.53c, Wayne Rasband, National Institutes of Health, USA) was utilised to measure VAT, SAT and WC. Total abdominal adipose tissue (TAT) area was calculated as the sum of visceral and SAT areas. Abdominal transverse diameter and sagittal diameter measurements were obtained from the abdomen images generated by the computer. Other covariates including age, height and weight were collected directly from the hospital electronic records.

### 2.3 Statistical Analysis

The analysis was conducted using R statistical software version 4.2.2. Missing values were addressed using multiple imputation, and a new dataset, obtained by averaging five imputed datasets, was employed for subsequent analysis. Descriptive statistics for baseline subject characteristics were reported as means and standard errors (SE). Differences between males and females were compared using Students' *t* tests or Wilcoxon rank‐sum tests depending on the data distribution. Effect sizes were reported with Cohen's d and Glass's Delta.

Initially, we constructed allometric growth models for each body measurement indicator based on 1054 healthy subjects. The age‐adjusted association between body composition with height can be described by the allometric model *Y* = *αX* ^*β*^ *Z* ^*γ*^, where *Y* represents the outcome, *X* denotes height, *β* is the scaling exponent (e.g., power), *Z* is the age covariate with power *γ* and *α* is the proportionality constant [14, 15]. The allometric growth model can be expressed in logarithmic form as log_e_ *Y* = log_e_ *α* + *β*log_e_ *X* + *γ*log_e_ *Z* + *ε*, where *ε* is the error term. After taking the logarithm of variables, the regression model was built with weight and other body composition measures (e.g., VAT and SAT) as the dependent variables and age and height as the independent variables. Five values: *α* (intercept), *β* (height), *γ* (age), *R* ^*2*^ and *P* value for each multiple regression model were presented in the results. Student's *t*‐tests or Wilcoxon rank‐sum tests were used to compare values of *β* of the regression models with the reference *β* value of 1.0 and 2.0. Simple linear regression analysis was performed between age‐adjusted partial correlation coefficients for the regression of body composition/height^*β*^ compared with height and the values of *β* in order to determine the scaling power independent of height, for each value of *β* ranging from 0.0 to 4.0, in increments of 0.1.

To further validate the clinical significance of the constructed indicators, we evaluated the relationships between these indicators and common chronic noncommunicable diseases including dyslipidemia, diabetes, cardio‐cerebrovascular diseases, cancers and abnormal bone mass by using a case–control study design. Healthy individuals served as the control group, providing a reference point for the analysis. Firstly, receiver operating characteristic curve (ROC) analyses were performed to determine the optimal cutoff value for body composition. Binary outputs were assigned as 0 if body composition measures were below the cutoff value and 1 if they were above the cutoff value. Binary logistic regression models were performed to evaluate the relationship between body composition/height^*β*^ and health outcomes. The main results adjusted for age, BMI and WC were presented using a forest plot. We also examined the relationships between unnormalised indicators and diseases. Additionally, considering the potential impact of multiple comorbidities, we performed a sensitivity analysis to examine the relationships between height‐normalised indices and individuals with only a single condition.

## 3 Results

### 3.1 Participant Characteristics

Table 1 displays the baseline characteristics of the 1054 healthy subjects, 644 (61.1%) of whom were male. In males, the mean age (± SE) was 45.412 ± 0.579 years, mean height was 170.205 ± 0.262 cm and mean weight was 66.655 ± 0.489 kg. For females, the mean age (± SE) was 42.393 ± 0.426 years, mean height was 159.169 ± 0.211 cm and mean weight was 55.130 ± 0.319 kg. Compared to females, males were significantly older (Δ: 3.019 ± 1.385 years; *p* < 0.001), taller (Δ: 11.035 ± 0.662 cm; *p* < 0.001) and heavier (Δ: 11.250 ± 1.250 kg; *p* < 0.001). Additionally, males had a higher BMI (Δ: 1.251 ± 0.360 kg/m^2^; *p* < 0.001), larger visceral adipose area (Δ: 27.797 ± 5.986 cm^2^; *p* < 0.001), greater waist circumference (Δ: 5.515 ± 1.054 cm; *p* < 0.001), increased transverse diameter (Δ: 1.159 ± 0.343 cm; *p* < 0.001), larger sagittal diameter (Δ: 2.018 ± 0.310 cm; *p* < 0.001) and smaller subcutaneous adipose area (Δ: −29.472 ± 6.625 cm^2^; *p* < 0.001). The characteristics of subjects with diseases are summarised in Table 2, with more detailed information available in Tables S1–S5.

**TABLE 1:** Characteristics of healthy subjects.

	Male (*n* = 410)	Female (*n* = 644)	Difference (Δ)	Standardised difference (d)
Age, years	45.412 ± 0.579	42.393 ± 0.426	3.019 ± 1.385^a^	0.270
Height, cm	170.205 ± 0.262	159.169 ± 0.211	11.035 ± 0.662^a^	2.068
Weight, kg	66.655 ± 0.489	55.130 ± 0.319	11.250 ± 1.250^a^	1.422
Body mass index, kg/m^2^	22.985 ± 0.151	21.734 ± 0.111	1.251 ± 0.360^a^	0.431
Visceral adipose area, cm^2^	91.840 ± 2.871	50.123 ± 1.393	27.797 ± 5.986^a^	1.180
Subcutaneous adipose area, cm^2^	110.187 ± 2.580	139.658 ± 2.135	−29.472 ± 6.625^a^	−0.551
Total abdominal adipose area, cm^2^	202.027 ± 4.971	198.782 ± 3.174	1.452 ± 11.415	0.040
Waist circumference, cm	85.642 ± 0.443	80.254 ± 0.322	5.515 ± 1.054^a^	0.660
Transverse diameter, cm	29.798 ± 0.133	28.639 ± 0.111	1.159 ± 0.343^a^	0.419
Sagittal diameter, cm	20.096 ± 0.134	18.039 ± 0.090	2.018 ± 0.310^a^	0.905

*Note:* All values are means ± standard errors.

^a^ *p* < 0.05.

**TABLE 2:** Characteristics of the subjects with diseases.

Characteristics	Subjects with dyslipidemia (*n* = 1159)	Subjects with type 2 diabetes (*n* = 803)	Subjects with cardio‐cerebrovascular diseases (*n* = 1289)	Subjects with cancers (*n* = 1108)	Subjects with abnormal bone mass (*n* = 509)
Age, years	46.476 ± 0.326	52.626 ± 0.344	54.387 ± 0.249	54.042 ± 0.258	65.668 ± 0.490
Height, cm	165.857 ± 0.226	164.999 ± 0.262	164.735 ± 0.210	163.859 ± 0.238	158.754 ± 0.351
Weight, kg	68.294 ± 0.363	66.802 ± 0.408	64.759 ± 0.337	61.972 ± 0.331	57.619 ± 0.418
Body mass index, kg/m^2^	24.735 ± 0.105	24.461 ± 0.120	23.787 ± 0.103	23.032 ± 0.104	22.827 ± 0.141
Visceral adipose area, cm^2^	124.445 ± 1.893	131.452 ± 2.388	115.606 ± 1.946	95.744 ± 1.830	114.890 ± 2.810
Subcutaneous adipose area, cm^2^	147.200 ± 1.842	140.631 ± 2.208	133.118 ± 1.809	126.341 ± 1.817	141.915 ± 2.663
Total abdominal adipose area, cm^2^	271.645 ± 3.101	272.083 ± 3.756	248.724 ± 3.198	222.085 ± 3.141	256.805 ± 4.733
Waist circumference, cm	90.155 ± 0.294	91.112 ± 0.346	88.844 ± 0.291	86.227 ± 0.295	86.957 ± 0.423
Transverse diameter, cm	31.225 ± 0.088	31.443 ± 0.105	30.804 ± 0.089	30.037 ± 0.091	30.385 ± 0.135
Sagittal diameter, cm	21.351 ± 0.093	21.648 ± 0.107	20.970 ± 0.090	20.246 ± 0.090	20.236 ± 0.127

*Note:* All values are presented as means ± standard errors.

### 3.2 Allometric Analyses

The results of allometric analyses are summarised in Table 3.

**TABLE 3:** Results of allometric growth model analyses.

	Male (*n* = 410)				Female (*n* = 644)			
	Power				Power			
	Height	Age	Intercept	*R* ^2^	Height	Age	Intercept	*R* ^2^
Weight, kg	2.037 ± 0.216^a^ ^–^ ^c^	0.022 ± 0.023	3.023 ± 0.159	0.178	2.203 ± 0.147^a^ ^–^ ^c^	0.127 ± 0.018	2.505 ± 0.103	0.276
Visceral adipose area, cm^2^	1.786 ± 1.270^a^, ^b^	0.472 ± 0.135	1.536 ± 0.935	0.025	1.274 ± 0.692^a^, ^b^	1.036 ± 0.085	−0.546 ± 0.485	0.186
Subcutaneous adipose area, cm^2^	2.266 ± 0.856^a^ ^–^ ^c^	0.056 ± 0.091	3.162 ± 0.630	0.012	1.656 ± 0.497^a^ ^–^ ^c^	0.411 ± 0.061	2.561 ± 0.349	0.070
Total abdominal adipose area, cm^2^	2.141 ± 0.967^a^ ^–^ ^c^	0.243 ± 0.103	3.102 ± 0.712	0.016	1.438 ± 0.489^a^ ^–^ ^c^	0.580 ± 0.060	2.383 ± 0.343	0.127
Waist circumference, cm	0.686 ± 0.166^a^ ^–^ ^c^	0.057 ± 0.018	3.863 ± 0.123	0.048	0.701 ± 0.110^a^ ^–^ ^c^	0.137 ± 0.014	3.547 ± 0.077	0.162
Transverse diameter, cm	0.688 ± 0.143^a^, ^b^	0.047 ± 0.015	2.848 ± 0.105	0.058	0.708 ± 0.106^a^, ^b^	0.127 ± 0.013	2.550 ± 0.074	0.157
Sagittal diameter, cm	0.646 ± 0.217^a^ ^–^ ^c^	0.050 ± 0.023	2.458 ± 0.160	0.022	0.678 ± 0.141^a^ ^–^ ^c^	0.143 ± 0.017	2.039 ± 0.099	0.109

*Note:* All values are means ± standard errors.

^a^ *p* < 0.05, compared with scaling power of 1.

^b^ *p* < 0.05, compared with scaling power of 2.

^c^ *p* < 0.05, comparing male vs. female.

#### 3.2.1 Body Weight

After adjustment for age, body weight scaled to height with a power of 2.307 ± 0.216 in males, which was significantly different from both 1 (*p* < 0.001) and 2 (*p* = 0.035). In females, body weight scaled to height with a power of 2.203 ± 0.147, which was significantly different from both 1 and 2 (all *p* < 0.001). In males, age was not a predictor of body weight (0.022 ± 0.023; *p* = 0.336), while in females, it is a positive predictor (0.127 ± 0.018; *p* < 0.001). The difference in powers between males and females was statistically significant (*p* < 0.001).

#### 3.2.2 VAT Area

After adjustment for age, VAT scaled to height with a power of 1.786 ± 1.270 in males, which was significantly different from both 1 (*p* < 0.001) and 2 (*p* = 0.023). In females, VAT scaled to height with a power of 1.274 ± 0.692, which was significantly different from 1 and 2 (all *p* < 0.001). Age was a positive predictor of VAT area in both males (0.472 ± 0.135; *p* < 0.001) and females (1.036 ± 0.085; *p* < 0.001). The difference in powers between males and females was not statistically significant (*p* = 0.077).

#### 3.2.3 SAT Area

After adjustment for age, SAT scaled to height with a power of 2.266 ± 0.856 in males and 1.656 ± 0.497 in females, which were both significantly different from 1 and 2 (all *p* < 0.001). In males, age was not a predictor of SAT area (0.056 ± 0.091; *p* = 0.537), while in females, it is a positive predictor (0.411 ± 0.061; *p* < 0.001). The difference in powers between males and females was statistically significant (*p* < 0.001).

#### 3.2.4 TAT Area

After adjustment for age, TAT scaled to height with a power of 2.141 ± 0.967 in males, which was significantly different from 1 (*p* < 0.001) and 2 (*p* = 0.030). In females, TAT scaled to height with a power of 1.438 ± 0.489, which was significantly different from 1 and 2 (all *p* < 0.001). Age was a positive predictor of TAT area in both males (0.243 ± 0.103; *p* = 0.019) and females (0.580 ± 0.060; *p* < 0.001). The difference in powers between males and females was statistically significant (*p* < 0.001).

#### 3.2.5 Waist Circumference

After adjustment for age, WC scaled to height with a power of 0.686 ± 0.166 in males and 0.701 ± 0.110 in females, which were both significantly different from 1 and 2 (all *p* < 0.001). Age was a positive predictor of waist circumference in both males (0.057 ± 0.018; *p* = 0.001) and females (0.137 ± 0.014; *p* < 0.001). The difference in powers between males and females was statistically significant (*p* < 0.001).

#### 3.2.6 Transverse Diameter

After adjustment for age, transverse diameter scaled to height with a power of 0.688 ± 0.143 in males and 0.708 ± 0.106 in females, which were both significantly different from 1 and 2 (all *p* < 0.001). Age was a positive predictor of transverse diameter in males (0.047 ± 0.015; *p* = 0.002) and females (0.127 ± 0.013; *p* < 0.001). The difference in powers between males and females was not statistically significant (*p* = 0.640).

#### 3.2.7 Sagittal Diameter

After adjustment for age, sagittal diameter scaled to height with a power of 0.646 ± 0.217 in males and 0.678 ± 0.141 in females, which were both significantly different from 1 and 2 (all *p* < 0.001). Age was a positive predictor of sagittal diameter in males (0.050 ± 0.023; *p* = 0.030) and females (0.143 ± 0.017; *p* < 0.001). The difference in powers between males and females was statistically significant (*p* < 0.001).

### 3.3 The Scaling Power of Body Composition Independent of Height

**FIGURE 1:**
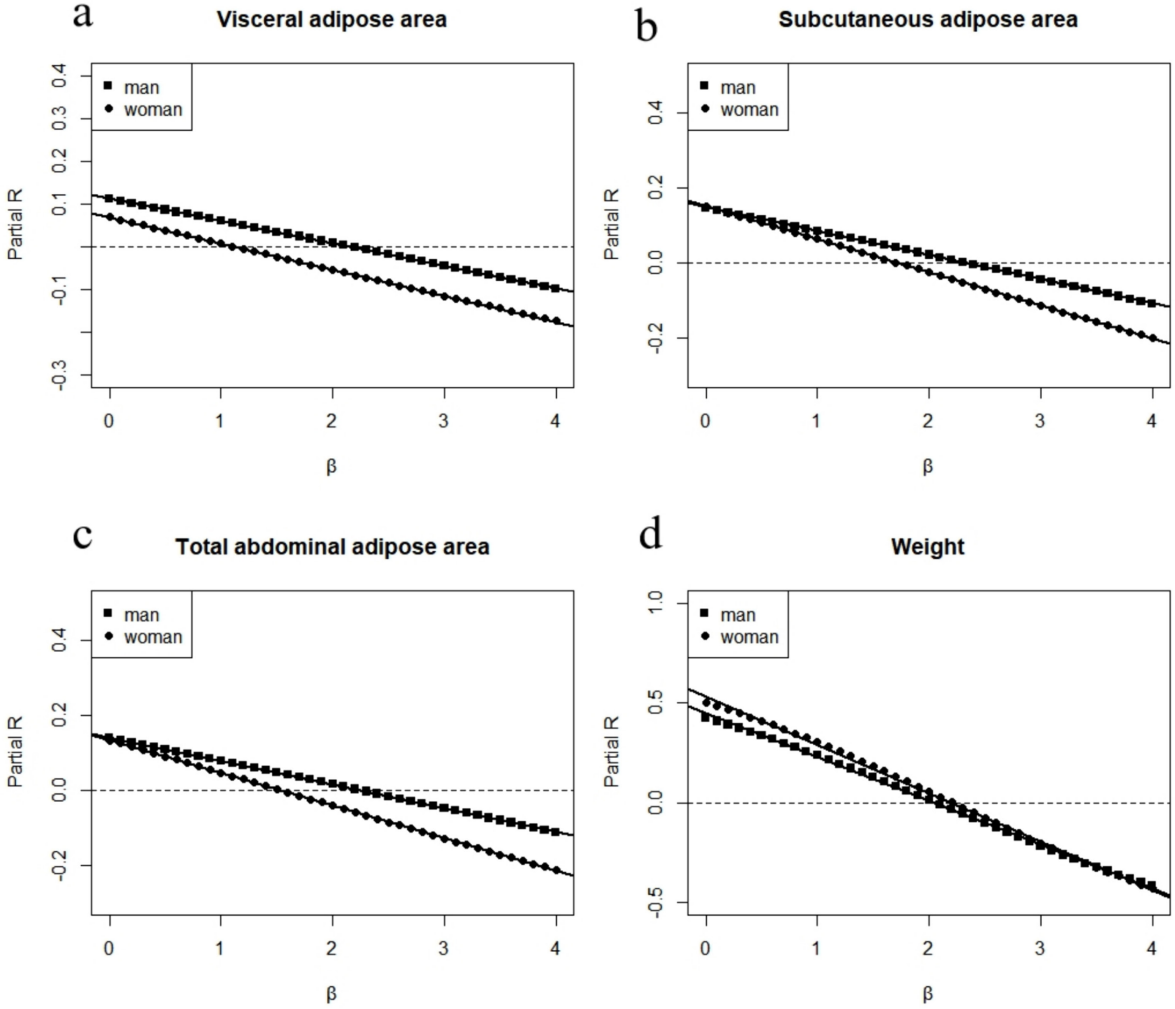
The age‐adjusted partial correlation coefficients between body composition/height^*β*^ and height at various scaling powers of *β* ranging from 0.0 to 4.0, for (a) visceral adipose area, (b) subcutaneous adipose area, (c) total abdominal adipose area and (d) weight.

Figure 1 illustrates the age‐adjusted partial correlation coefficients between body composition/height^*β*^ and height at various scaling powers of *β* (ranging from 0.0 to 4.0). When scaling VAT to height, a power of 2.173 for males and 1.115 for females resulted in independence of height. Simple linear regression analysis was used to generate the following lines:y = −0.052x + 0.113 (*R* ^*2*^ = 0.9999, *p* < 0.001) for males and y = −0.061x + 0.068 (*R* ^*2*^ = 0.9999, *p* < 0.001) for females.

For SAT, a power of 2.313 for males and 1.716 for females was recommended when scaling to height. Simple linear regression analysis was used to generate the following lines:y = −0.064x + 0.148 (*R* ^*2*^ = 0.9999, *p* < 0.001) for males and y = −0.088x + 0.151 (*R* ^*2*^ = 1, *p* < 0.001) for females.

For TAT, a power of 2.238 for males and 1.540 for females was recommended when scaling to height. Simple linear regression analysis was used to generate the following lines:y = −0.063x + 0.141 (*R* ^*2*^ = 0.9999, *p* < 0.001) for males and y = −0.087x + 0.134 (*R* ^*2*^ = 1, *p* < 0.001) for females.

For weight, a power of 2.037 for males and 2.193 for females was recommended when scaling to height. Simple linear regression analysis was used to generate the following lines:y = −0.219x + 0.446 (*R* ^*2*^ = 0.9991, *p* < 0.001) for males and y = −0.243x + 0.533 (*R* ^*2*^ = 0.9984, *p* < 0.001) for females. Additional results for other indicators are shown in Figure S2.

### 3.4 Association Between Body Composition/Height^*β*^ and Comorbidities

**FIGURE 2:**
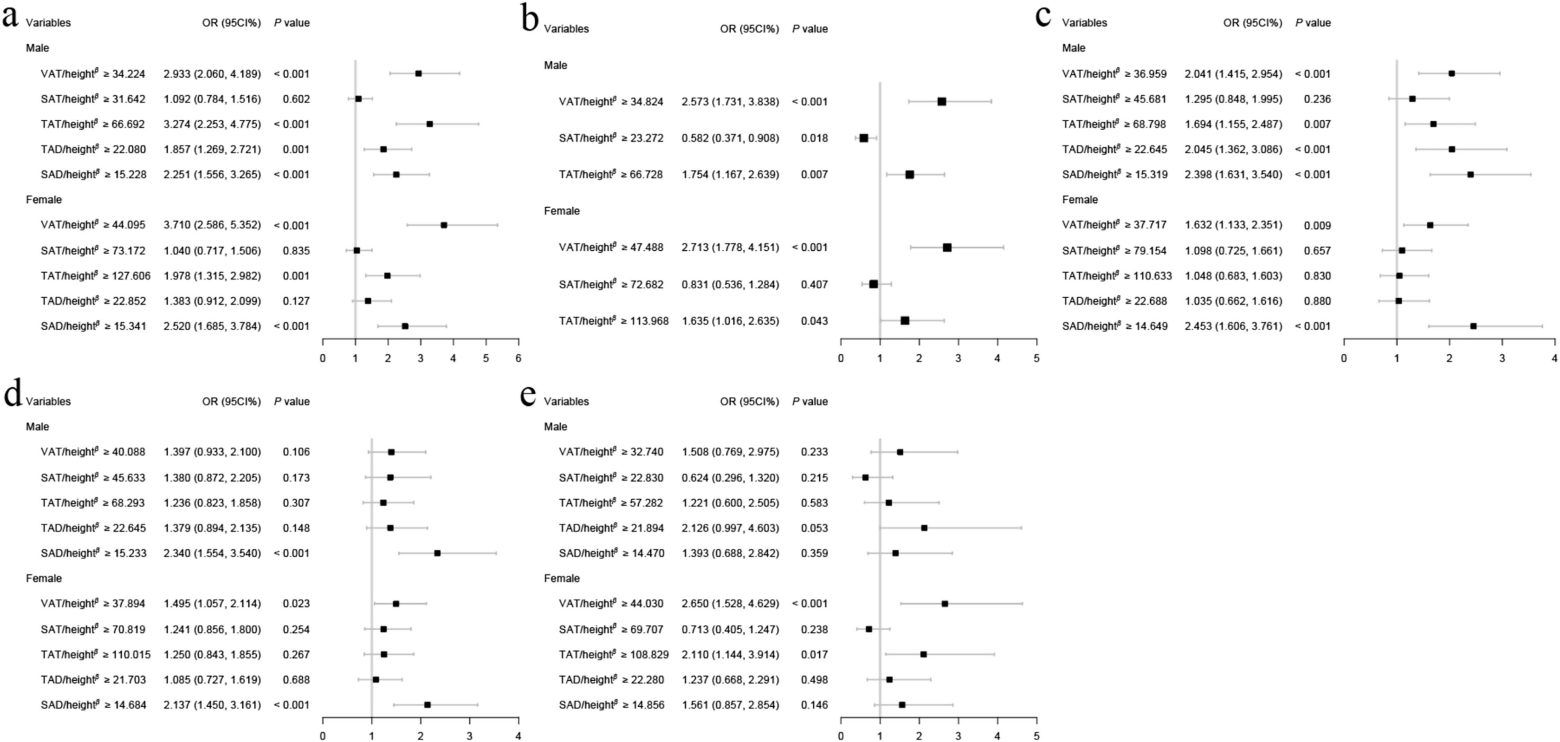
Association of height‐normalised body composition indicators with (a) dyslipidemia, (b) type 2 diabetes, (c) cardio‐cerebrovascular diseases, (d) cancers and (e) abnormal bone mass.

Across all analysed health conditions, the ratio of body composition to height (body composition/height^*β*^) consistently showed slightly superior area under the curve (AUC) values when compared to body composition alone, regardless of gender. These results can be found in Tables S6–S10. Figure 2 highlights the relationship between body composition/height^*β*^ and comorbidities in both men and women after adjusting for age, BMI and WC. The detailed information is provided in Tables S11–S15. Significant associations were maintained between VAT/height^*β*^, TAT/height^*β*^ and SAD/height^*β*^ and dyslipidemia in both genders. In the context of type 2 diabetes, significant associations were retained for both VAT/height^*β*^ and TAT/height^*β*^ across both genders. Similarly, in cardio‐cerebrovascular diseases, significant associations were preserved for both VAT/height^*β*^ and SAD/height^*β*^ in men and women. For cancers, SAD/height^*β*^ retained its significant association in both genders. Additionally, VAT/height^*β*^ maintained a significant association with abnormal bone mass, but only in women. The data suggest that VAT/height^*β*^, TAT/height^*β*^ and SAD/height^*β*^ have strong and consistent associations with these diseases.

Given the potential for confounding effects due to multiple comorbidities, we conducted a sensitivity analysis. The detailed information about subjects with multiple comorbidities is provided in Tables S16 and S17. We excluded individuals with multiple comorbidities, so that only those with a single condition were analysed. The results were presented in Tables S18–S22. These results confirm that the associations were largely consistent even after controlling for potential confounding by multiple comorbidities, suggesting that such interactions had minimal impact on the observed associations.

## 4 Discussion

Our research introduces a significant advancement in the field of abdominal adiposity assessment through the development and validation of height‐normalised indices—body composition/height^*β*^. By using the allometric growth model, we eliminated the effect of height on the body composition measures, rather than simply taking the ratio of the body composition to height. These novel height‐normalised indices outperformed their respective body composition indices in predictive performance. Our findings underscore the potential clinical value of these new indicators in health outcomes assessment, stressing the importance of incorporating height‐normalised indices into clinical practice.

By addressing the limitations of traditional body composition measurements, which fail to capture individual variations in body size and composition, our study underscores the importance of height in the evaluation of abdominal adiposity. By incorporating height into our novel indices, we provide a more accurate and significant assessment of the associations of health outcomes with abdominal fat. Our research emphasises the need to factor in height when evaluating a person's abdominal adiposity and associated health outcomes. The novel VAT/height^*β*^, TAT/height^*β*^ and SAD/height^*β*^ indices we have developed not only enable a more precise assessment of abdominal adiposity but also reveal stronger associations with diseases. Our results show that both height‐normalised and nonnormalised adiposity indices (Tables S23–S27) are significantly associated with comorbidities. However, the associations tend to be more pronounced with height normalisation, highlighting its utility in providing a refined analysis of how adiposity affects health outcomes across different body sizes.

In analysing the relationships between the indices and health outcomes, we observed significant correlations with increased odds of dyslipidemia, diabetes, cardio‐cerebrovascular diseases and cancers in both sexes. Notably, it has been suggested that SAD is a more effective indicator of abdominal visceral adiposity than WC, displaying stronger correlations [16, 17]. This finding is supported by the use of CT scans in our study for accurate quantification of adiposity, further supporting the superior predictive value of SAD for cardiovascular and metabolic risks, which is consistent with previous studies [16, 18, 19]. Although our study employed CT scans to measure SAD, conventional methods typically involve an inexpensive, straightforward procedure using an abdominal calliper to measure the distance between the dorsum and the apex of the abdomen, which is measured at the midpoint between the iliac crest and the last rib in the supine position [20]. This measurement is very convenient, as is the measurement of height, and further underscores the value of SAD as an indicator in assessing abdominal fat and health outcomes.

However, our study did not find a significant association between SAT/height^*β*^ and comorbidities, highlighting the need for further research into the distinct roles of different adipose tissues in metabolic health. This gap underscores the complexity of the impact of adiposity on health and the need for targeted investigation of the underlying mechanisms.

Our findings broaden and enhance the scope of previous studies that primarily focused on conventional body composition indicators, like BMI and WC [21, 22, 23]. By presenting height‐normalised abdominal adiposity indices, we provide a more exhaustive understanding of the relationships between body composition and health outcomes. The remarkable associations observed emphasise the potential clinical utility of these new indices, suggesting that they could improve health outcomes assessment and guide the development of personalised intervention strategies.

Although BMI is significantly associated with several comorbidities, it does not distinguish between fat and muscle mass, nor does it accurately reflect fat distribution. Our study examined more specific adiposity indices like VAT and SAT, which provide clearer insights into fat distribution on health outcomes. Moreover, we found that VAT/height^*β*^ was positively associated with abnormal bone mass in women, while BMI showed a negative association in men. The nuances revealed by height‐normalised indices highlight the critical need for these additional measures. Subgroup analyses by BMI (Tables S28–S32) showed that VAT/height^*β*^ consistently demonstrated significant associations with chronic diseases across BMI categories and sexes. Its pronounced impact in lower BMI categories suggested visceral fat played a significant role even at lower BMI levels, highlighting its importance for early prevention and intervention strategies.

While the large sample size and comprehensive evaluation of body composition measures underscore the strengths of our study, its cross‐sectional design limits the ability to infer causality between the indices and diseases. Prospective studies are required to verify these associations and to examine the predictive capability of these indices. In addition, the applicability of our findings to diverse populations warrants further investigation, given the focus of our study on a Chinese population.

In conclusion, our study established height‐normalised abdominal adiposity indices that hold potential usefulness in the clinical evaluation of abdominal adiposity. These indices incorporate the scaling relationships between height and abdominal adiposity, demonstrating significant associations with health outcomes in both males and females. Our results underline the importance of body composition in health outcomes assessment and propose that these new indices may facilitate enhanced risk stratification. Further research is necessary to validate these indices across various populations and investigate their potential applicability in clinical practice.

## Ethics Statement

This study was approved by the medical ethics committee of the Second Affiliated Hospital of Wenzhou Medical University (2022‐K‐306‐01, Figure S3). As we utilised only anonymous clinical data, the study did not require informed consent from participants.

## Conflicts of Interest

The authors declare no conflicts of interest.

## Supporting information


**Figure S1.** Flow Diagram of Participants Selection.
**Figure S2.** The age‐adjusted partial correlation coefficients between body composition/height^*β*^ and height at various scaling powers of β ranging from 0.0 to 4.0, for (a) waist circumference, (b) transverse diameter and (c) sagittal diameter. For waist circumference, a power of 0.626 for males and 0.605 for females was recommended when scaling to height. Simple linear regression analysis was used to generate the following lines:y = −0.235x + 0.147 (R^2^ = 0.9808, *p* < 0.001) for males and y = −0.256x + 0.155 (R^2^ = 0.9685, *p* < 0.001) for females. For transverse diameter, a power of 0.613 for males and 0.617 for females was recommended when scaling to height. Simple linear regression analysis was used to generate the following lines:y = −0.256x + 0.157 (R^2^ = 0.9713, *p* < 0.001) for males and y = −0.261x + 0.161 (R^2^ = 0.9672, *p* < 0.001) for females. For sagittal diameter, a power of 0.607 for males and 0.595 for females was recommended when scaling to height. Simple linear regression analysis was used to generate the following lines:y = −0.196x + 0.119 (R^2^ = 0.9912, *p* < 0.001) for males and y = −0.220x + 0.131 (R^2^ = 0.9835, *p* < 0.001) for females.
**Figure S3.** The ethics committee approval file.
**Table S1.** Characteristics of subjects with dyslipidemia.
**Table S2.** Characteristics of subjects with type 2 diabetes.
**Table S3.** Characteristics of subjects with cardio‐cerebrovascular diseases.
**Table S4.** Characteristics of subjects with cancers.
**Table S5.** Characteristics of subjects with abnormal bone mass.
**Table S6.** Thresholds of body composition indicators for dyslipidemia by gender.
**Table S7.** Thresholds of body composition indicators for type 2 diabetes by gender.
**Table S8.** Thresholds of body composition indicators for cardio‐cerebrovascular diseases by gender.
**Table S9.** Thresholds of body composition indicators for cancers by gender.
**Table S10.** Thresholds of body composition indicators for abnormal bone mass by gender.
**Table S11.** Association of height‐normalised body composition indicators with dyslipidemia.
**Table S12.** Association of height‐normalised body composition indicators with type 2 diabetes.
**Table S13.** Association of height‐normalised body composition indicators with cardio‐cerebrovascular diseases.
**Table S14.** Association of height‐normalised body composition indicators with cancers.
**Table S15.** Association of height‐normalised body composition indicators with abnormal bone mass.
**Table S16.** Patient distribution by number and combination of comorbidities.
**Table S17.** Patient distribution by number of comorbidities.
**Table S18.** Association of height‐normalised body composition indicators with dyslipidemia (*N* = 718).
**Table S19.** Association of height‐normalised body composition indicators with type 2 diabetes (*N* = 239).
**Table S20.** Association of height‐normalised body composition indicators with cardio‐cerebrovascular diseases (*N* = 494).
**Table S21.** Association of height‐normalizsed body composition indicators with cancers (*N* = 738).
**Table S22.** Association of height‐normalised body composition indicators with abnormal bone mass (*N* = 210).
**Table S23.** Association of unnormalised body composition indicators with dyslipidemia.
**Table S24.** Association of unnormalised body composition indicators with type 2 diabetes.
**Table S25.** Association of unnormalised body composition indicators with cardio‐cerebrovascular diseases.
**Table S26.** Association of unnormalised body composition indicators with cancers.
**Table S27.** Association of unnormalised body composition indicators with abnormal bone mass.
**Table S28–1.** Association of height‐normalised body composition indicators with dyslipidemia stratified by BMI in males.
**Table S28–2.** Association of height‐normalised body composition indicators with dyslipidemia stratified by BMI in females.
**Table S29–1.** Association of height‐normalised body composition indicators with type 2 diabetes stratified by BMI in males.
**Table S29–2.** Association of height‐normalised body composition indicators with type 2 diabetes stratified by BMI in females.
**Table S30–1.** Association of height‐normalised body composition indicators with cardio‐cerebrovascular diseases stratified by BMI in males.
**Table S30–2.** Association of height‐normalised body composition indicators with cardio‐cerebrovascular diseases stratified by BMI in females.
**Table 31–1.** Association of height‐normalised body composition indicators with cancers stratified by BMI in males.
**Table S31–2.** Association of height‐normalised body composition indicators with cancers stratified by BMI in females.
**Table S32–1.** Association of height‐normalised body composition indicators with abnormal bone mass stratified by BMI in males.
**Table S32–2.** Association of height‐normalised body composition indicators with abnormal bone mass stratified by BMI in females.

## Data Availability

Raw data were generated at the Second Affiliated Hospital of Wenzhou Medical University. The data underlying this article will be shared on reasonable request to the corresponding author.

## References

[1] G. Eknoyan , “Adolphe Quetelet (1796 1874) the Average Man and Indices of Obesity,” Nephrology, Dialysis, Transplantation 23, no. 1 (2008): 47–51. 10.1093/ndt/gfm517 17890752

[2] K. J. Rothman , “BMI‐Related Errors in the Measurement of Obesity,” International Journal of Obesity 32, no. Suppl 3 (2008): S56–S59. 18695655 10.1038/ijo.2008.87

[3] D. J. Rader , “Effect of Insulin Resistance, Dyslipidemia, and Intra‐Abdominal Adiposity on the Development of Cardiovascular Disease and Diabetes Mellitus,” The American Journal of Medicine 120, no. 3 Suppl 1 (2007): S12–S18. 10.1016/j.amjmed.2007.01.003 17320517

[4] M. C. Carr and J. D. Brunzell , “Abdominal Obesity and Dyslipidemia in the Metabolic Syndrome: Importance of Type 2 Diabetes and Familial Combined Hyperlipidemia in Coronary Artery Disease Risk,” The Journal of Clinical Endocrinology and Metabolism 89, no. 6 (2004): 2601–2607. 15181030 10.1210/jc.2004-0432

[5] E. J. Rhee , “The Influence of Obesity and Metabolic Health on Vascular Health,” Endocrinology and Metabolism (Seoul) 37, no. 1 (2022): 1–8. 10.3803/EnM.2022.101 PMC8901957 35255597

[6] K. Hidayat , X. Du , G. Chen , M. Shi , and B. Shi , “Abdominal Obesity and Lung Cancer Risk: Systematic Review and Meta‐Analysis of Prospective Studies,” Nutrients 8, no. 12 (2016): 810. 27983672 10.3390/nu8120810 PMC5188465

[7] S. S. Jayant , R. Gupta , A. Rastogi , et al., “Abdominal Obesity and Incident Cardio‐Metabolic Disorders in Asian‐Indians: A 10‐Years Prospective Cohort Study,” Diabetes and Metabolic Syndrome: Clinical Research and Reviews 16, no. 2 (2022): 102418. 10.1016/j.dsx.2022.102418 35123378

[8] H. Fang , E. Berg , X. Cheng , and W. Shen , “How to Best Assess Abdominal Obesity,” Current Opinion in Clinical Nutrition and Metabolic Care 21, no. 5 (2018): 360–365. 29916924 10.1097/MCO.0000000000000485 PMC6299450

[9] A. Shuster , M. Patlas , J. H. Pinthus , and M. Mourtzakis , “The Clinical Importance of Visceral Adiposity: A Critical Review of Methods for Visceral Adipose Tissue Analysis,” The British Journal of Radiology 85, no. 1009 (2012): 1–10. 21937614 10.1259/bjr/38447238 PMC3473928

[10] H. H. Hu , K. S. Nayak , and M. I. Goran , “Assessment of Abdominal Adipose Tissue and Organ Fat Content by Magnetic Resonance Imaging,” Obesity Reviews 12, no. 5 (2011): e504–e515. 21348916 10.1111/j.1467-789X.2010.00824.x PMC3079791

[11] Q. Zeng , L. Wang , S. Dong , et al., “CT‐Derived Abdominal Adiposity: Distributions and Better Predictive Ability Than BMI in a Nationwide Study of 59,429 Adults in China,” Metabolism 115 (2021): 154456. 33259834 10.1016/j.metabol.2020.154456

[12] M. Kong , M. Xu , Y. Zhou , et al., “Assessing Visceral Obesity and Abdominal Adipose Tissue Distribution in Healthy Populations Based on Computed Tomography: A Large Multicenter Cross‐Sectional Study,” Frontiers in Nutrition 9 (2022): 871697. 35548570 10.3389/fnut.2022.871697 PMC9082940

[13] S. B. Heymsfield , Z. Wang , R. N. Baumgartner , and R. Ross , “Human Body Composition: Advances in Models and Methods,” Annual Review of Nutrition 17 (1997): 527–558. 10.1146/annurev.nutr.17.1.527 9240939

[14] P. Kaitaniemi , “Testing the Allometric Scaling Laws,” Journal of Theoretical Biology 228, no. 2 (2004): 149–153. 15094011 10.1016/j.jtbi.2003.12.007

[15] J. C. Brown , S. B. Heymsfield , and B. J. Caan , “Scaling of Computed Tomography Body Composition to Height: Relevance of Height‐Normalized Indices in Patients With Colorectal Cancer,” Journal of Cachexia, Sarcopenia and Muscle 13, no. 1 (2022): 203–209. 34741439 10.1002/jcsm.12847 PMC8818649

[16] A. M. Madden and S. Smith , “Body Composition and Morphological Assessment of Nutritional Status in Adults: A Review of Anthropometric Variables,” Journal of Human Nutrition and Dietetics 29, no. 1 (2016): 7–25. 10.1111/jhn.12278 25420774

[17] P. Pajunen , H. Rissanen , M. A. Laaksonen , M. Heliövaara , A. Reunanen , and P. Knekt , “Sagittal Abdominal Diameter as a New Predictor for Incident Diabetes,” Diabetes Care 36, no. 2 (2013): 283–288. 22961578 10.2337/dc11-2451 PMC3554316

[18] G. Valsamakis , A. Jones , R. Chetty , et al., “MRI Total Sagittal Abdominal Diameter as a Predictor of Metabolic Syndrome Compared to Visceral Fat at L4‐L5 Level,” Current Medical Research and Opinion 24, no. 7 (2008): 1853–1860. 18507894 10.1185/03007990802185757

[19] H. S. Kahn , Q. Gu , K. M. Bullard , D. S. Freedman , N. Ahluwalia , and C. L. Ogden , “Population Distribution of the Sagittal Abdominal Diameter (SAD) From a Representative Sample of US Adults: Comparison of SAD, Waist Circumference and Body Mass Index for Identifying Dysglycemia,” PLoS ONE 9, no. 10 (2014): e108707. 25272003 10.1371/journal.pone.0108707 PMC4182731

[20] H. S. Kahn , H. Austin , D. F. Williamson , and D. Arensberg , “Simple Anthropometric Indices Associated With Ischemic Heart Disease,” Journal of Clinical Epidemiology 49, no. 9 (1996): 1017–1024. 8780611 10.1016/0895-4356(96)00113-8

[21] M. Heo , G. C. Kabat , D. Gallagher , S. B. Heymsfield , and T. E. Rohan , “Optimal Scaling of Weight and Waist Circumference to Height for Maximal Association With DXA‐Measured Total Body Fat Mass by Sex, Age and Race/Ethnicity,” International Journal of Obesity 37, no. 8 (2013): 1154–1160. 23207404 10.1038/ijo.2012.201

[22] S. B. Heymsfield , C. M. Peterson , D. M. Thomas , et al., “Scaling of Adult Body Weight to Height Across Sex and Race/Ethnic Groups: Relevance to BMI,” The American Journal of Clinical Nutrition 100, no. 6 (2014): 1455–1461. 25411280 10.3945/ajcn.114.088831 PMC4232013

[23] K. Hood , J. Ashcraft , K. Watts , et al., “Allometric Scaling of Weight to Height and Resulting Body Mass Index Thresholds in Two Asian Populations,” Nutrition & Diabetes 9, no. 1 (2019): 2. 30683839 10.1038/s41387-018-0068-3 PMC6347591

