# Supplementary material for: Evaluation of Health Associations With Height‐Normalised Abdominal Body Composition Indices: A Single‐Centre Cross‐Sectional Study
Source: J Cachexia Sarcopenia Muscle. 2024 Oct 7;15(6):2651–9. doi: 10.1002/jcsm.13609 (PMC11634505; doi:10.1002/jcsm.13609)
Supplement: Supplementary file 1 — Figure S1. Flow Diagram of Participants Selection. Figure S2. The age‐adjusted partial correlation coefficients between body composition/height β and height at various scaling powers of β ranging from 0.0 to 4.0, for (a) waist circumference, (b) transverse diameter and (c) sagittal diameter. For waist circumference, a power of 0.626 for males and 0.605 for females was recommended when scaling to height. Simple linear regression analysis was used to generate the following lines:y = −0.235x + 0.147 (R2 = 0.9808, p < 0.001) for males and y = −0.256x + 0.155 (R2 = 0.9685, p < 0.001) for females. For transverse diameter, a power of 0.613 for males and 0.617 for females was recommended when scaling to height. Simple linear regression analysis was used to generate the following lines:y = −0.256x + 0.157 (R2 = 0.9713, p < 0.001) for males and y = −0.261x + 0.161 (R2 = 0.9672, p < 0.001) for females. For sagittal diameter, a power of 0.607 for males and 0.595 for females was recommended when scaling to height. Simple linear regression analysis was used to generate the following lines:y = −0.196x + 0.119 (R2 = 0.9912, p < 0.001) for males and y = −0.220x + 0.131 (R2 = 0.9835, p < 0.001) for females. Figure S3. The ethics committee approval file. Table S1. Characteristics of subjects with dyslipidemia. Table S2. Characteristics of subjects with type 2 diabetes. Table S3. Characteristics of subjects with cardio‐cerebrovascular diseases. Table S4. Characteristics of subjects with cancers. Table S5. Characteristics of subjects with abnormal bone mass. Table S6. Thresholds of body composition indicators for dyslipidemia by gender. Table S7. Thresholds of body composition indicators for type 2 diabetes by gender. Table S8. Thresholds of body composition indicators for cardio‐cerebrovascular diseases by gender. Table S9. Thresholds of body composition indicators for cancers by gender. Table S10. Thresholds of body composition indicators for abnormal bone mass by gender. Table S11. A [file JCSM-15-2651-s001.docx]

**Supplementary Materials**


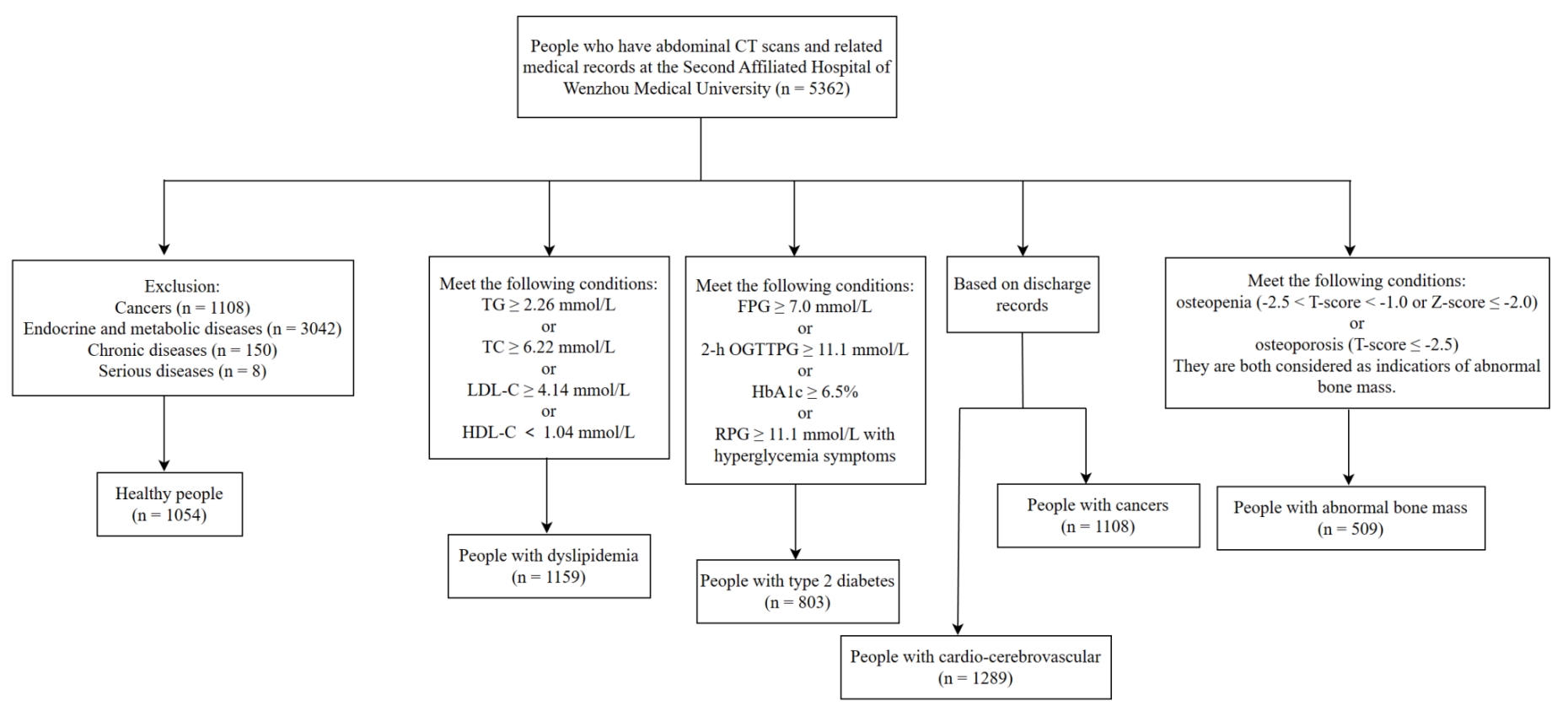


**Figure S1** Flow Diagram of **P**articipants **Selection**


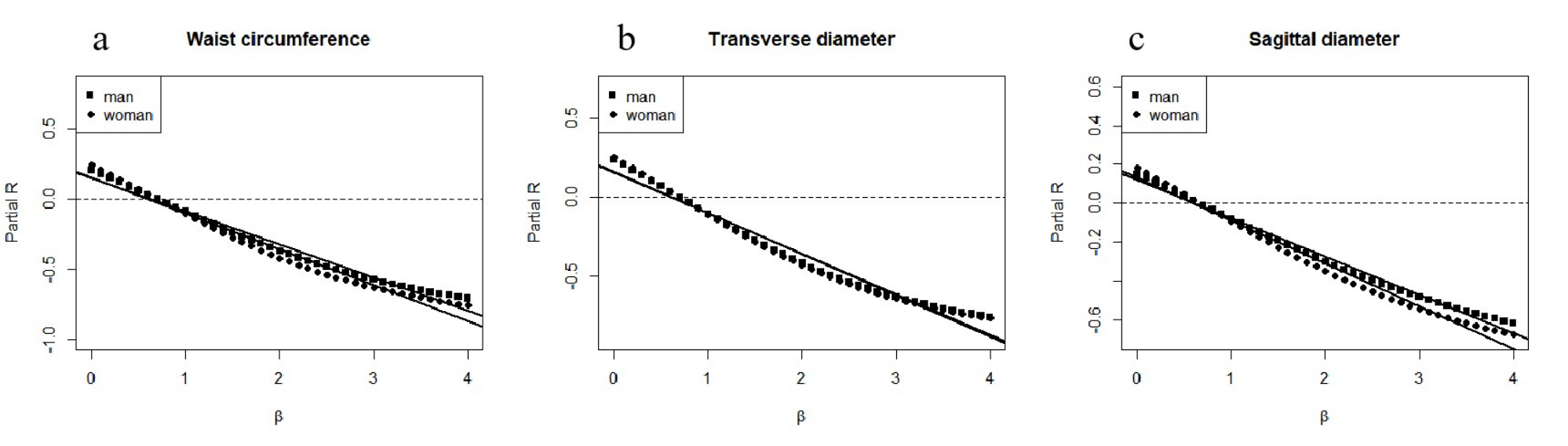


**Figure S2** The age-adjusted partial correlation coefficients between body composition/height*^β^* and height at various scaling powers of β ranging from 0.0 to 4.0, for **(a)** waist circumference, **(b)** transverse diameter and **(c)** sagittal diameter.

For waist circumference, a power of 0.626 for males and 0.605 for females was recommended when scaling to height. Simple linear regression analysis was used to generate the following lines: y = -0.235x + 0.147 (R^2^ = 0.9808, *P* < 0.001) for males and y = -0.256x + 0.155 (R^2^ = 0.9685, *P* < 0.001) for females. For transverse diameter, a power of 0.613 for males and 0.617 for females was recommended when scaling to height. Simple linear regression analysis was used to generate the following lines: y = -0.256x + 0.157 (R^2^ = 0.9713, *P* < 0.001) for males and y = -0.261x + 0.161 (R^2^ = 0.9672, *P* < 0.001) for females. For sagittal diameter, a power of 0.607 for males and 0.595 for females was recommended when scaling to height. Simple linear regression analysis was used to generate the following lines: y = -0.196x + 0.119 (R^2^ = 0.9912, *P* < 0.001) for males and y = -0.220x + 0.131 (R^2^ = 0.9835, *P* < 0.001) for females.


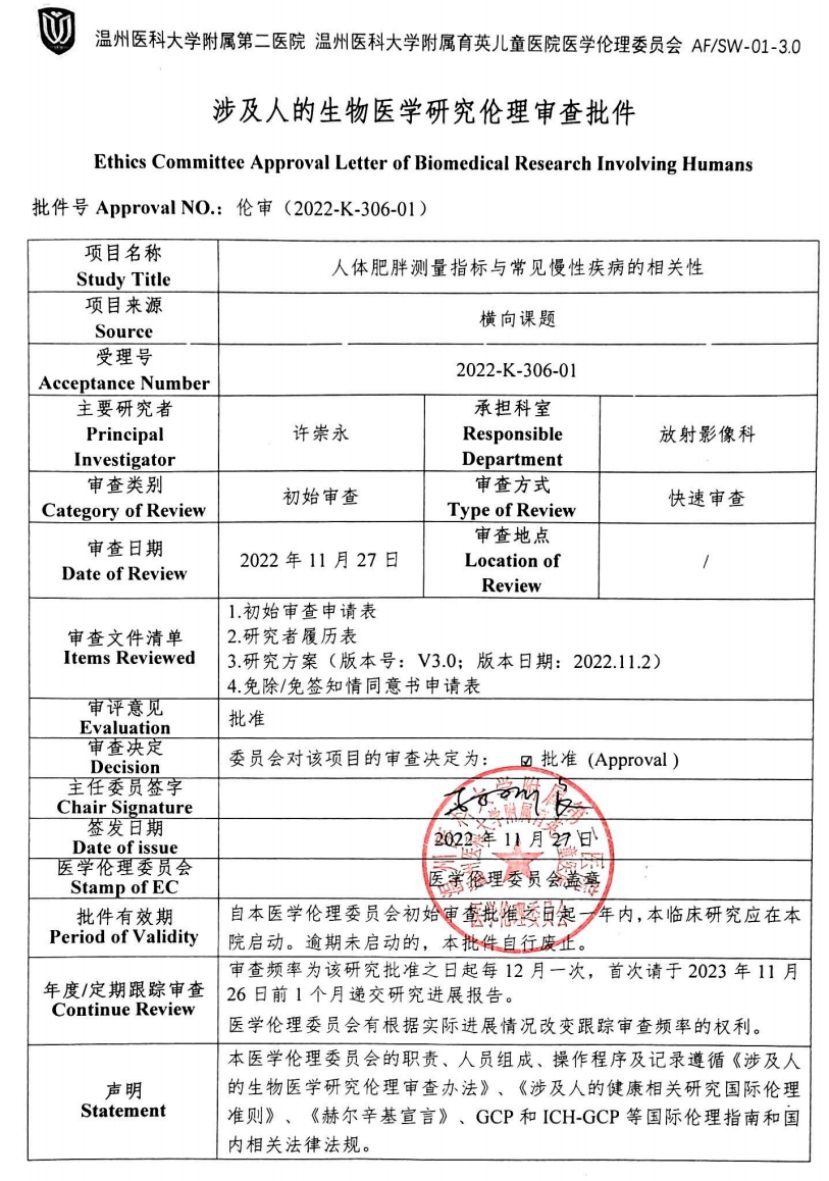


**Figure S3** The **E**thics **C**ommittee **A**pproval **File**

**Table S1** Characteristics of **S**ubjects with **D**yslipidemia

|  | Male (*n* = 761) | Female (*n* = 398) | Difference (Δ) | Standardized difference (d) |
| --- | --- | --- | --- | --- |
| Age, years | 46.503 ± 0.396 | 46.425 ± 0.572 | 0.079 ± 1.347 | 0.007 |
| Height, cm | 169.790 ± 0.207 | 158.338 ± 0.246 | 11.500 ± 0.500^a^ | 2.337 |
| Weight, kg | 72.475 ± 0.416 | 60.299 ± 0.494 | 12.250 ± 1.250^a^ | 1.236 |
| Body mass index, kg/m^2^ | 25.098 ± 0.126 | 24.040 ± 0.185 | 1.058 ± 0.432^a^ | 0.297 |
| Visceral adipose area, cm² | 139.671 ± 2.386 | 95.331 ± 2.517 | 43.931 ± 6.990^a^ | 0.883 |
| Subcutaneous adipose area, cm² | 134.721 ± 2.071 | 171.062 ± 3.305 | -34.307 ± 6.887^a^ | -0.551 |
| Total abdominal adipose area, cm^2^ | 274.392 ± 3.885 | 266.394 ± 5.133 | 7.998 ± 12.811 | 0.076 |
| Waist circumference, cm | 92.072 ± 0.350 | 86.489 ± 0.487 | 5.584 ± 1.174^a^ | 0.577 |
| Transverse diameter, cm | 31.620 ± 0.104 | 30.470 ± 0.156 | 1.150 ± 0.359^a^ | 0.389 |
| Sagittal diameter, cm | 22.078 ± 0.110 | 19.959 ± 0.146 | 2.119 ± 0.364^a^ | 0.707 |

All values are means ± standard errors.

^a^ *P* < 0.05.

**Table S2** Characteristics of **S**ubjects with **T**ype 2 **D**iabetes

|  | Male (*n* = 515) | Female (*n* = 288) | Difference (Δ) | Standardized difference (d) |
| --- | --- | --- | --- | --- |
| Age, years | 52.445 ± 0.419 | 52.951 ± 0.597 | -0.507 ± 1.407 | -0.052 |
| Height, cm | 168.938 ± 0.238 | 157.955 ± 0.292 | 10.983 ± 0.757^a^ | 2.095 |
| Weight, kg | 70.492 ± 0.483 | 60.205 ± 0.558 | 10.100 ± 1.400^a^ | 1.086 |
| Body mass index, kg/m^2^ | 24.658 ± 0.148 | 24.108 ± 0.206 | 0.550 ± 0.492^a^ | 0.162 |
| Visceral adipose area, cm^2^ | 145.299 ± 3.173 | 106.689 ± 2.979 | 36.839 ± 9.000^a^ | 0.764 |
| Subcutaneous adipose area, cm^2^ | 121.689 ± 2.245 | 174.505 ± 3.951 | -52.064 ± 8.266^a^ | -0.788 |
| Total abdominal adipose area, cm^2^ | 266.988 ± 4.766 | 281.194 ± 6.058 | -14.206 ± 15.349 | -0.134 |
| Waist circumference, cm | 92.510 ± 0.423 | 88.612 ± 0.571 | 3.898 ± 1.390^a^ | 0.405 |
| Transverse diameter, cm | 31.676 ± 0.126 | 31.026 ± 0.187 | 0.645 ± 0.453^a^ | 0.204 |
| Sagittal diameter, cm | 22.232 ± 0.133 | 20.604 ± 0.161 | 1.628 ± 0.422^a^ | 0.557 |

All values are means ± standard errors.

^a^ *P* < 0.05.

**Table S3** Characteristics of **S**ubjects with **C**ardio-**C**erebrovascular **D**iseases

|  | Male (*n* = 832) | Female (*n* = 457) | Difference (Δ) | Standardized difference (d) |
| --- | --- | --- | --- | --- |
| Age, years | 54.124 ± 0.308 | 54.867 ± 0.421 | -0.743 ± 1.020 | -0.083 |
| Height, cm | 168.528 ± 0.199 | 157.828 ± 0.239 | 10.700 ± 0.700^a^ | 2.091 |
| Weight, kg | 68.108 ± 0.408 | 58.664 ± 0.472 | 9.350 ± 1.350^a^ | 0.936 |
| Body mass index, kg/m^2^ | 23.930 ± 0.127 | 23.527 ± 0.174 | 0.404 ± 0.420 | 0.109 |
| Visceral adipose area, cm^2^ | 127.115 ± 2.622 | 94.654 ± 2.427 | 29.363 ± 7.776^a^ | 0.626 |
| Subcutaneous adipose area, cm^2^ | 115.798 ± 1.936 | 164.649 ± 3.204 | -47.630 ± 6.623^a^ | -0.713 |
| Total abdominal adipose area, cm^2^ | 242.913 ± 4.108 | 259.303 ± 5.009 | -17.254 ± 13.078^a^ | -0.153 |
| Waist circumference, cm | 90.235 ± 0.360 | 86.313 ± 0.474 | 3.922 ± 1.176^a^ | 0.381 |
| Transverse diameter, cm | 31.043 ± 0.107 | 30.369 ± 0.155 | 0.674 ± 0.363^a^ | 0.212 |
| Sagittal diameter, cm | 21.552 ± 0.112 | 19.911 ± 0.137 | 1.635 ± 0.352^a^ | 0.562 |

All values are means ± standard errors.

^a^ *P* < 0.05.

**Table S4** Characteristics of **S**ubjects with **C**ancers

|  | Male (*n* = 632) | Female (*n* = 476) | Difference (Δ) | Standardized difference (d) |
| --- | --- | --- | --- | --- |
| Age, years | 55.098 ± 0.322 | 52.641 ± 0.411 | 2.000 ± 1.000^a^ | 0.274 |
| Height, cm | 168.704 ± 0.224 | 157.427 ± 0.257 | 11.277 ± 0.670^a^ | 2.004 |
| Weight, kg | 66.017 ± 0.409 | 56.602 ± 0.438 | 9.415 ±1.188^a^ | 0.944 |
| Body mass index, kg/m^2^ | 23.181 ± 0.133 | 22.835 ± 0.167 | 0.451 ± 0.409^a^ | 0.095 |
| Visceral adipose area, cm^2^ | 107.244 ± 2.705 | 80.475 ± 2.099 | 22.390 ± 6.940^a^ | 0.584 |
| Subcutaneous adipose area, cm^2^ | 106.362 ± 1.999 | 152.867 ± 2.874 | -45.605 ± 6.430^a^ | -0.742 |
| Total abdominal adipose area, cm^2^ | 213.606 ± 4.328 | 233.342 ± 4.476 | -21.076 ± 12.309^a^ | -0.202 |
| Waist circumference, cm | 88.042 ± 0.388 | 83.816 ± 0.432 | 4.226 ± 1.144^a^ | 0.440 |
| Transverse diameter, cm | 30.341 ± 0.115 | 29.633 ± 0.144 | 0.741 ± 0.356^a^ | 0.225 |
| Sagittal diameter, cm | 21.014 ± 0.120 | 19.228 ± 0.123 | 1.805 ± 0.347^a^ | 0.667 |

All values are means ± standard errors.

^a^ *P* < 0.05.

**Table S5** Characteristics of **S**ubjects with **A**bnormal **B**one **M**ass

|  | Male (*n* = 154) | Female (*n* = 355) | Difference (Δ) | Standardized difference (d) |
| --- | --- | --- | --- | --- |
| Age, years | 64.318 ± 0.913 | 66.254 ± 0.579 | -1.935 ± 2.092 | -0.175 |
| Height, cm | 166.645 ± 0.469 | 155.330 ± 0.321 | 11.314 ± 1.134^a^ | 1.891 |
| Weight, kg | 63.297 ± 0.707 | 55.156 ± 0.458 | 8.140 ± 1.643^a^ | 0.939 |
| Body mass index, kg/m^2^ | 22.783 ± 0.235 | 22.846 ± 0.175 | -0.063 ± 0.603 | -0.020 |
| Visceral adipose area, cm^2^ | 129.076 ± 6.225 | 108.736 ± 2.938 | 16.173 ± 13.021^a^ | 0.367 |
| Subcutaneous adipose area, cm^2^ | 108.310 ± 3.604 | 156.493 ± 3.189 | -46.365 ± 9.831^a^ | -0.802 |
| Total abdominal adipose area, cm^2^ | 237.386 ± 9.168 | 265.229 ± 5.447 | -27.843 ± 20.115^a^ | -0.262 |
| Waist circumference, cm | 88.693 ± 0.766 | 86.204 ± 0.503 | 2.900 ± 1.799^a^ | 0.262 |
| Transverse diameter, cm | 30.572 ± 0.228 | 30.304 ± 0.166 | 0.269 ± 0.578 | 0.088 |
| Sagittal diameter, cm | 21.118 ± 0.242 | 19.853 ± 0.145 | 1.265 ± 0.534^a^ | 0.449 |

All values are means ± standard errors.

^a^ *P* < 0.05.

**Table S6** Thresholds of **B**ody **C**omposition **Indicators for D**yslipidemia **by Gender**

|  | Male | | | | Female | | | |
| --- | --- | --- | --- | --- | --- | --- | --- | --- |
|  | Threshold | AUC | 95% CI | *P* value | Threshold | AUC | 95% CI | *P* value |
| Weight | 68.2 | 0.656 | (0.623, 0.688) | < 0.001 | 57.01 | 0.658 | (0.623, 0.692) | < 0.001 |
| BMI | 24 | 0.679 | (0.648, 0.711) | < 0.001 | 24 | 0.688 | (0.655, 0.722) | < 0.001 |
| VAT | 112.026 | 0.716 | (0.685, 0.747) | < 0.001 | 77.414 | 0.723 | (0.690, 0.756) | < 0.001 |
| VAT/height*^β^* | 34.224 | 0.717 | (0.686, 0.748) | < 0.001 | 44.095 | 0.725 | (0.692, 0.758) | < 0.001 |
| SAT | 109.970 | 0.639 | (0.605, 0.672) | < 0.001 | 152.005 | 0.646 | (0.612, 0.681) | < 0.001 |
| SAT/height*^β^* | 31.642 | 0.644 | (0.611, 0.677) | < 0.001 | 73.172 | 0.652 | (0.617, 0.686) | < 0.001 |
| TAT | 227.084 | 0.701 | (0.669, 0.732) | < 0.001 | 244.269 | 0.702 | (0.669, 0.735) | < 0.001 |
| TAT/height*^β^* | 66.692 | 0.704 | (0.673, 0.735) | < 0.001 | 127.606 | 0.707 | (0.674, 0.74) | < 0.001 |
| WC | 86.952 | 0.699 | (0.668, 0.729) | < 0.001 | 87.250 | 0.690 | (0.657, 0.724) | < 0.001 |
| WC/height*^β^* | 63.194 | 0.704 | (0.673, 0.734) | < 0.001 | 64.509 | 0.698 | (0.665, 0.732) | < 0.001 |
| TAD | 31.047 | 0.690 | (0.659, 0.722) | < 0.001 | 31.291 | 0.673 | (0.639, 0.707) | < 0.001 |
| TAD/height*^β^* | 22.080 | 0.697 | (0.666, 0.728) | < 0.001 | 22.852 | 0.682 | (0.648, 0.716) | < 0.001 |
| SAD | 20.960 | 0.694 | (0.663, 0.725) | < 0.001 | 19.130 | 0.697 | (0.664, 0.730) | < 0.001 |
| SAD/height*^β^* | 15.228 | 0.697 | (0.666, 0.728) | < 0.001 | 15.341 | 0.703 | (0.670, 0.736) | < 0.001 |

**Receiver operating characteristic (ROC) curve analyses were performed to determine the optimal cutoffs of the indices, as shown in the table.** BMI, body mass index; VAT, visceral adipose tissue area; SAT, subcutaneous adipose tissue area; TAT, total abdominal adipose tissue area; WC, waist circumference; TAD, transverse diameter; SAD, sagittal diameter; AUC, area under the curve; 95% CI, 95% confidence interval.

**Table S7** Thresholds of **B**ody **C**omposition **Indicators for T**ype 2 **D**iabetes **by Gender**

|  | Male | | | | Female | | | |
| --- | --- | --- | --- | --- | --- | --- | --- | --- |
|  | Threshold | AUC | 95% CI | *P* value | Threshold | AUC | 95% CI | *P* value |
| Weight | 68.45 | 0.605 | (0.568, 0.641) | < 0.001 | 57.55 | 0.662 | (0.624, 0.701) | < 0.001 |
| BMI | 24 | 0.645 | (0.61, 0.681) | < 0.001 | 24 | 0.703 | (0.666, 0.740) | < 0.001 |
| VAT | 112.351 | 0.724 | (0.691, 0.756) | < 0.001 | 82.618 | 0.783 | (0.750, 0.816) | < 0.001 |
| VAT/height*^β^* | 34.824 | 0.730 | (0.697, 0.762) | < 0.001 | 47.488 | 0.786 | (0.753, 0.819) | < 0.001 |
| SAT | 97.125 | 0.568 | (0.530, 0.605) | < 0.001 | 161.242 | 0.662 | (0.624, 0.701) | < 0.001 |
| SAT/height*^β^* | 23.272 | 0.577 | (0.540, 0.614) | < 0.001 | 72.682 | 0.671 | (0.632, 0.709) | < 0.001 |
| TAT | 227.392 | 0.677 | (0.642, 0.711) | < 0.001 | 240.849 | 0.743 | (0.708, 0.779) | < 0.001 |
| TAT/height*^β^* | 66.728 | 0.686 | (0.652, 0.720) | < 0.001 | 113.968 | 0.749 | (0.714, 0.784) | < 0.001 |
| WC | 87.948 | 0.707 | (0.673, 0.740) | < 0.001 | 85.634 | 0.750 | (0.715, 0.785) | < 0.001 |
| WC/height*^β^* | 63.088 | 0.721 | (0.688, 0.754) | < 0.001 | 64.652 | 0.761 | (0.727, 0.795) | < 0.001 |
| TAD | - | 1 | - | < 0.001 | - | 1 | - | < 0.001 |
| TAD/height*^β^* | - | 1 | - | < 0.001 | - | 1 | - | < 0.001 |
| SAD | - | 1 | - | < 0.001 | - | 1 | - | < 0.001 |
| SAD/height*^β^* | - | 1 | - | < 0.001 | - | 1 | - | < 0.001 |

**Receiver operating characteristic (ROC) curve analyses were performed to determine the optimal cutoffs of the indices, as shown in the table.** In the context of diabetes, both TAD (TAD/height*^β^*) and SAD (SAD/height*^β^*) demonstrated an AUC of 1 for both genders, signifying their perfect distinction between diabetic and healthy individuals. BMI, body mass index; VAT, visceral adipose tissue area; SAT, subcutaneous adipose tissue area; TAT, total abdominal adipose tissue area; WC, waist circumference; TAD, transverse diameter; SAD, sagittal diameter; AUC, area under the curve; 95% CI, 95% confidence interval.

**Table S8** Thresholds of **B**ody **C**omposition **Indicators for** **C**ardio-**C**erebrovascular **D**iseases **by Gender**

|  | Male | | | | Female | | | |
| --- | --- | --- | --- | --- | --- | --- | --- | --- |
|  | Threshold | AUC | 95% CI | *P* value | Threshold | AUC | 95% CI | *P* value |
| Weight | 68.95 | 0.536 | (0.503, 0.569) | 0.019 | 58.85 | 0.604 | (0.569, 0.638) | < 0.001 |
| BMI | 24 | 0.576 | (0.543, 0.609) | < 0.001 | 24 | 0.647 | (0.613, 0.680) | < 0.001 |
| VAT | 120.224 | 0.639 | (0.608, 0.670) | < 0.001 | 65.341 | 0.712 | (0.680, 0.743) | < 0.001 |
| VAT/height*^β^* | 36.959 | 0.648 | (0.616, 0.679) | < 0.001 | 37.717 | 0.716 | (0.684, 0.746) | < 0.001 |
| SAT | 60.309 | 0.524 | (0.490, 0.558) | 0.085 | 167.429 | 0.612 | (0.577, 0.646) | < 0.001 |
| SAT/height*^β^* | 45.681 | 0.539 | (0.505, 0.573) | 0.013 | 79.154 | 0.621 | (0.587, 0.655) | < 0.001 |
| TAT | 227.222 | 0.603 | (0.57, 0.635) | < 0.001 | 240.849 | 0.674 | (0.641, 0.707) | < 0.001 |
| TAT/height*^β^* | 68.798 | 0.615 | (0.583, 0.647) | < 0.001 | 110.633 | 0.682 | (0.649, 0.714) | < 0.001 |
| WC | 87.027 | 0.634 | (0.603, 0.666) | < 0.001 | 86.926 | 0.680 | (0.647, 0.712) | < 0.001 |
| WC/height*^β^* | 63.842 | 0.652 | (0.621, 0.683) | < 0.001 | 65.280 | 0.693 | (0.661, 0.725) | < 0.001 |
| TAD | 30.897 | 0.621 | (0.589, 0.653) | < 0.001 | 30.087 | 0.655 | (0.622, 0.688) | < 0.001 |
| TAD/height*^β^* | 22.645 | 0.641 | (0.610, 0.673) | < 0.001 | 22.688 | 0.670 | (0.637, 0.702) | < 0.001 |
| SAD | 20.953 | 0.683 | (0.606, 0.670) | < 0.001 | 19.270 | 0.690 | (0.658, 0.723) | < 0.001 |
| SAD/height*^β^* | 15.319 | 0.650 | (0.618, 0.682) | < 0.001 | 14.649 | 0.701 | (0.669, 0.733) | < 0.001 |

**Receiver operating characteristic (ROC) curve analyses were performed to determine the optimal cutoffs of the indices, as shown in the table.** BMI, body mass index; VAT, visceral adipose tissue area; SAT, subcutaneous adipose tissue area; TAT, total abdominal adipose tissue area; WC, waist circumference; TAD, transverse diameter; SAD, sagittal diameter; AUC, area under the curve; 95% CI, 95% confidence interval.

**Table S9** Thresholds of **B**ody **C**omposition **Indicators for C**ancers **by Gender**

|  | Male | | | | Female | | | |
| --- | --- | --- | --- | --- | --- | --- | --- | --- |
|  | Threshold | AUC | 95% CI | *P* value | Threshold | AUC | 95% CI | *P* value |
| Weight | 84.7 | 0.483 | (0.447, 0.518) | 0.829 | 59.75 | 0.540 | (0.505, 0.574) | 0.011 |
| BMI | 24 | 0.514 | (0.479, 0.55) | 0.218 | 24 | 0.585 | (0.551, 0.620) | < 0.001 |
| VAT | 120.195 | 0.563 | (0.528, 0.599) | < 0.001 | 65.303 | 0.644 | (0.611, 0.677) | < 0.001 |
| VAT/height*^β^* | 40.088 | 0.571 | (0.536, 0.606) | < 0.001 | 37.894 | 0.648 | (0.616, 0.681) | < 0.001 |
| SAT | 59.903 | 0.479 | (0.443, 0.514) | 0.880 | 156.256 | 0.567 | (0.533, 0.602) | < 0.001 |
| SAT/height*^β^* | 45.633 | 0.491 | (0.455, 0.527) | 0.692 | 70.819 | 0.580 | (0.546, 0.614) | < 0.001 |
| TAT | 226.134 | 0.529 | (0.494, 0.565) | 0.056 | 235.870 | 0.609 | (0.575, 0.643) | < 0.001 |
| TAT/height*^β^* | 68.293 | 0.540 | (0.504, 0.575) | 0.015 | 110.015 | 0.619 | (0.586, 0.653) | < 0.001 |
| WC | 87.148 | 0.573 | (0.538, 0.608) | < 0.001 | 87.647 | 0.612 | (0.579, 0.646) | < 0.001 |
| WC/height*^β^* | 63.790 | 0.588 | (0.553, 0.623) | < 0.001 | 62.301 | 0.629 | (0.596, 0.662) | < 0.001 |
| TAD | 31.420 | 0.553 | (0.517, 0.588) | 0.002 | 28.501 | 0.595 | (0.561, 0.629) | < 0.001 |
| TAD/height*^β^* | 22.645 | 0.569 | (0.534, 0.604) | < 0.001 | 21.703 | 0.613 | (0.580, 0.647) | < 0.001 |
| SAD | 21.509 | 0.591 | (0.556, 0.626) | < 0.001 | 19.140 | 0.628 | (0.595, 0.661) | < 0.001 |
| SAD/height*^β^* | 15.233 | 0.601 | (0.566, 0.636) | < 0.001 | 14.684 | 0.640 | (0.607, 0.673) | < 0.001 |

**Receiver operating characteristic (ROC) curve analyses were performed to determine the optimal cutoffs of the indices, as shown in the table.** BMI, body mass index; VAT, visceral adipose tissue area; SAT, subcutaneous adipose tissue area; TAT, total abdominal adipose tissue area; WC, waist circumference; TAD, transverse diameter; SAD, sagittal diameter; AUC, area under the curve; 95% CI, 95% confidence interval.

**Table S10** Thresholds of **B**ody **C**omposition **Indicators for A**bnormal **B**one **M**ass **by Gender**

|  | Male | | | | Female | | | |
| --- | --- | --- | --- | --- | --- | --- | --- | --- |
|  | Threshold | AUC | 95% CI | *P* value | Threshold | AUC | 95% CI | *P* value |
| Weight | 63.55 | 0.596 | (0.544, 0.648) | 1.000 | 59.8 | 0.505 | (0.467, 0.543) | 0.396 |
| BMI | 24 | 0.472 | (0.419, 0.524) | 0.851 | 24 | 0.598 | (0.561, 0.635) | < 0.001 |
| VAT | 123.033 | 0.644 | (0.590, 0.697) | < 0.001 | 74.361 | 0.775 | (0.744, 0.807) | < 0.001 |
| VAT/height*^β^* | 32.740 | 0.660 | (0.607, 0.712) | < 0.001 | 44.030 | 0.783 | (0.752, 0.814) | < 0.001 |
| SAT | 52.181 | 0.498 | (0.445, 0.55) | 0.537 | 146.225 | 0.587 | (0.550, 0.624) | < 0.001 |
| SAT/height*^β^* | 22.830 | 0.529 | (0.476, 0.582) | 0.148 | 69.707 | 0.614 | (0.578, 0.651) | < 0.001 |
| TAT | 243.684 | 0.590 | (0.536, 0.644) | < 0.001 | 240.683 | 0.700 | (0.665, 0.735) | < 0.001 |
| TAT/height*^β^* | 57.282 | 0.615 | (0.561, 0.668) | < 0.001 | 108.829 | 0.719 | (0.684, 0.753) | < 0.001 |
| WC | 86.822 | 0.593 | (0.539, 0.647) | < 0.001 | 83.540 | 0.686 | (0.651, 0.721) | < 0.001 |
| WC/height*^β^* | 62.677 | 0.626 | (0.574, 0.679) | < 0.001 | 63.899 | 0.720 | (0.686, 0.753) | < 0.001 |
| TAD | 31.439 | 0.578 | (0.523, 0.632) | 0.002 | 29.312 | 0.661 | (0.626, 0.697) | < 0.001 |
| TAD/height*^β^* | 21.894 | 0.614 | (0.560, 0.667) | < 0.001 | 22.280 | 0.697 | (0.663, 0.731) | < 0.001 |
| SAD | 20.711 | 0.599 | (0.546, 0.653) | < 0.001 | 19.149 | 0.693 | (0.658, 0.728) | < 0.001 |
| SAD/height*^β^* | 14.470 | 0.626 | (0.574, 0.678) | < 0.001 | 14.856 | 0.719 | (0.685, 0.752) | < 0.001 |

**Receiver operating characteristic (ROC) curve analyses were performed to determine the optimal cutoffs of the indices, as shown in the table.** BMI, body mass index; VAT, visceral adipose tissue area; SAT, subcutaneous adipose tissue area; TAT, total abdominal adipose tissue area; WC, waist circumference; TAD, transverse diameter; SAD, sagittal diameter; AUC, area under the curve; 95% CI, 95% confidence interval.

**Table S11 Association of Height-Normalized Body Composition Indicators with D**yslipidemia

|  | Crude OR (95% CI) | *P* value | Adj. OR (95% CI)^1^ | *P* value | Adj. OR (95% CI)^2^ | *P* value | Adj. OR (95% CI)^3^ | *P* value |
| --- | --- | --- | --- | --- | --- | --- | --- | --- |
| Male |  |  |  |  |  |  |  |  |
| BMI ≥ 24 | 3.087 (2.407, 3.971) | < 0.001 | - | - | 1.410 (0.997, 1.993) | 0.052 | - | - |
| VAT/height*^β^* ≥ 34.224 | 4.603 (3.567, 5.962) | < 0.001 | 3.196 (2.302, 4.454) | < 0.001 | 2.967 (2.086, 4.235) | < 0.001 | 2.933 (2.060, 4.189) | < 0.001 |
| SAT/height*^β^* ≥ 31.642 | 2.557 (1.998, 3.279) | < 0.001 | 1.295 (0.947, 1.769) | 0.105 | 1.121 (0.807, 1.553) | 0.495 | 1.092 (0.784, 1.516) | 0.602 |
| TAT/height*^β^* ≥ 66.692 | 4.876 (3.773, 6.323) | < 0.001 | 3.521 (2.499, 4.982) | < 0.001 | 3.318 (2.287, 4.834) | < 0.001 | 3.274 (2.253, 4.775) | < 0.001 |
| WC/height*^β^* ≥ 63.194 | 4.112 (3.194, 5.311) | < 0.001 | 2.632 (1.850, 3.757) | < 0.001 | - | - | - | - |
| TAD/height*^β^* ≥ 22.080 | 3.740 (2.910, 4.822) | < 0.001 | 2.203 (1.560, 3.118) | < 0.001 | 1.898 (1.300, 2.775) | < 0.001 | 1.857 (1.269, 2.721) | 0.001 |
| SAD/height*^β^* ≥ 15.228 | 4.013 (3.115, 5.192) | < 0.001 | 2.544 (1.828, 3.553) | < 0.001 | 2.262 (1.564, 3.279) | < 0.001 | 2.251 (1.556, 3.265) | < 0.001 |
| Female |  |  |  |  |  |  |  |  |
| BMI ≥ 24 | 3.605 (2.732, 4.774) | < 0.001 | - | - | 1.654 (1.128, 2.426) | 0.010 | - | - |
| VAT/height*^β^* ≥ 44.095 | 5.683 (4.336, 7.484) | < 0.001 | 3.669 (2.594, 5.216) | < 0.001 | 3.955 (2.766, 5.686) | < 0.001 | 3.710 (2.586, 5.352) | < 0.001 |
| SAT/height*^β^* ≥ 73.172 | 2.807 (2.165, 3.646) | < 0.001 | 1.169 (0.829, 1.644) | 0.370 | 1.111 (0.770, 1.601) | 0.571 | 1.040 (0.717, 1.506) | 0.835 |
| TAT/height*^β^* ≥ 127.606 | 4.254 (3.242, 5.603) | < 0.001 | 2.026 (1.398, 2.942) | < 0.001 | 2.133 (1.425, 3.202) | < 0.001 | 1.978 (1.315, 2.982) | 0.001 |
| WC/height*^β^* ≥ 64.509 | 4.253 (3.255, 5.577) | < 0.001 | 2.049 (1.404, 2.995) | < 0.001 | - | - | - | - |
| TAD/height*^β^* ≥ 22.852 | 3.596 (2.756, 4.705) | < 0.001 | 1.511 (1.043, 2.188) | 0.029 | 1.485 (0.984, 2.243) | 0.060 | 1.383 (0.912, 2.099) | 0.127 |
| SAD/height*^β^* ≥ 15.341 | 4.839 (3.639, 6.470) | < 0.001 | 2.482 (1.719, 3.593) | < 0.001 | 2.644 (1.773, 3.959) | < 0.001 | 2.520 (1.685, 3.784) | < 0.001 |

BMI, body mass index; VAT, visceral adipose tissue area; SAT, subcutaneous adipose tissue area; TAT, total abdominal adipose tissue area; WC, waist circumference; TAD, transverse diameter; SAD, sagittal diameter; OR, odds ratio; 95% CI, 95% confidence interval.

^1^ Adjustment for age and BMI.

^2^ Adjustment for age and WC.

^3^ Adjustment for age, BMI and WC.

**Table S12** **Association of Height-Normalized Body Composition Indicators with T**ype 2 **D**iabetes

|  | Crude OR (95% CI) | *P* value | Adj. OR (95% CI)^1^ | *P* value | Adj. OR (95% CI)^2^ | *P* value | Adj. OR (95% CI)^3^ | *P* value |
| --- | --- | --- | --- | --- | --- | --- | --- | --- |
| Male |  |  |  |  |  |  |  |  |
| BMI ≥ 24 | 2.319 (1.778, 3.033) | < 0.001 | - | - | 0.849 (0.570, 1.260) | 0.418 | - | - |
| VAT/height*^β^* ≥ 34.824 | 4.949 (3.748, 6.567) | < 0.001 | 3.606 (2.509, 5.214) | < 0.001 | 2.516 (1.695, 3.749) | < 0.001 | 2.573 (1.731, 3.838) | < 0.001 |
| SAT/height*^β^* ≥ 23.272 | 2.126 (1.542, 2.942) | < 0.001 | 0.920 (0.607, 1.390) | 0.693 | 0.566 (0.361, 0.880) | 0.012 | 0.582 (0.371, 0.908) | 0.018 |
| TAT/height*^β^* ≥ 66.728 | 4.003 (3.045, 5.285) | < 0.001 | 2.670 (1.846, 3.875) | < 0.001 | 1.698 (1.131, 2.552) | 0.011 | 1.754 (1.167, 2.639) | 0.007 |
| WC/height*^β^* ≥ 63.088 | 4.158 (3.159, 5.496) | < 0.001 | 2.798 (1.907, 4.122) | < 0.001 | - | - | - | - |
| Female |  |  |  |  |  |  |  |  |
| BMI ≥ 24 | 4.104 (3.033, 5.570) | < 0.001 | - | - | 1.208 (0.775, 1.876) | 0.402 | - | - |
| VAT/height*^β^* ≥ 47.488 | 7.603 (5.590, 10.419) | < 0.001 | 3.380 (2.263, 5.073) | < 0.001 | 2.665 (1.755, 4.057) | < 0.001 | 2.713 (1.778, 4.151) | < 0.001 |
| SAT/height*^β^* ≥ 72.682 | 3.276 (2.458, 4.380) | < 0.001 | 1.292 (0.866, 1.922) | 0.208 | 0.835 (0.539, 1.288) | 0.417 | 0.831 (0.536, 1.284) | 0.407 |
| TAT/height*^β^* ≥ 113.968 | 5.717 (4.229, 7.784) | < 0.001 | 2.419 (1.584, 3.703) | < 0.001 | 1.631 (1.016, 2.620) | 0.043 | 1.635 (1.016, 2.635) | 0.043 |
| WC/height*^β^* ≥ 64.652 | 6.413 (4.742, 8.725) | < 0.001 | 2.911 (1.886, 4.508) | < 0.001 | - | - | - | - |

The AUC values of 1 for both TAD (TAD/height*^β^*) and SAD (SAD/height*^β^*) in both genders suggest flawless distinction between different samples across all thresholds. In such cases, logistic regression models for binary classification can't yield meaningful coefficient estimates or odds ratios due to the perfect discrimination. BMI, body mass index; VAT, visceral adipose tissue area; SAT, subcutaneous adipose tissue area; TAT, total abdominal adipose tissue area; WC, waist circumference; OR, odds ratio; 95% CI, 95% confidence interval.

^1^ Adjustment for age and BMI.

^2^ Adjustment for age and WC.

^3^ Adjustment for age, BMI and WC.

**Table S13 Association of Height-Normalized Body Composition Indicators with C**ardio-**C**erebrovascular **D**iseases

|  | Crude OR (95% CI) | *P* value | Adj. OR (95% CI)^1^ | *P* value | Adj. OR (95% CI)^2^ | *P* value | Adj. OR (95% CI)^3^ | *P* value |
| --- | --- | --- | --- | --- | --- | --- | --- | --- |
| Male |  |  |  |  |  |  |  |  |
| BMI ≥ 24 | 1.709 (1.340, 2.184) | < 0.001 | - | - | 0.907 (0.630, 1.303) | 0.597 | - | - |
| VAT/height*^β^* ≥ 36.959 | 3.067 (2.384, 3.964) | < 0.001 | 2.657 (1.894, 3.749) | < 0.001 | 1.984 (1.379, 2.862) | < 0.001 | 2.041 (1.415, 2.954) | < 0.001 |
| SAT/height*^β^* ≥ 45.681 | 1.674 (1.216, 2.333) | 0.002 | 1.517 (1.003, 2.319) | 0.051 | 1.155 (0.764, 1.763) | 0.499 | 1.295 (0.848, 1.995) | 0.236 |
| TAT/height*^β^* ≥ 68.798 | 2.487 (1.950, 3.179) | < 0.001 | 2.302 (1.619, 3.287) | < 0.001 | 1.584 (1.085, 2.314) | 0.017 | 1.694 (1.155, 2.487) | 0.007 |
| WC/height*^β^* ≥ 63.842 | 2.778 (2.172, 3.565) | < 0.001 | 2.809 (1.933, 4.106) | < 0.001 | - | - | - | - |
| TAD/height*^β^* ≥ 22.645 | 2.691 (2.083, 3.494) | < 0.001 | 2.775 (1.909, 4.064) | < 0.001 | 1.892 (1.267, 2.837) | 0.002 | 2.045 (1.362, 3.086) | < 0.001 |
| SAD/height*^β^* ≥ 15.319 | 3.019 (2.356, 3.886) | < 0.001 | 3.141 (2.213, 4.487) | < 0.001 | 2.339 (1.595, 3.445) | < 0.001 | 2.398 (1.631, 3.540) | < 0.001 |
| Female |  |  |  |  |  |  |  |  |
| BMI ≥ 24 | 3.271 (2.500, 4.295) | < 0.001 | - | - | 1.660 (1.109, 2.490) | 0.014 | - | - |
| VAT/height*^β^* ≥ 37.717 | 4.432 (3.429, 5.754) | < 0.001 | 1.761 (1.243, 2.494) | 0.001 | 1.653 (1.151, 2.376) | 0.007 | 1.632 (1.133, 2.351) | 0.009 |
| SAT/height*^β^* ≥ 79.154 | 2.684 (2.059, 3.508) | < 0.001 | 1.276 (0.870, 1.873) | 0.212 | 1.126 (0.747, 1.696) | 0.571 | 1.098 (0.725, 1.661) | 0.657 |
| TAT/height*^β^* ≥ 110.633 | 3.239 (2.526, 4.166) | < 0.001 | 1.265 (0.866, 1.845) | 0.223 | 1.073 (0.703, 1.636) | 0.742 | 1.048 (0.683, 1.603) | 0.830 |
| WC/height*^β^* ≥ 65.280 | 4.106 (3.156, 5.363) | < 0.001 | 1.889 (1.268, 2.822) | 0.002 | - | - | - | - |
| TAD/height*^β^* ≥ 22.688 | 3.106 (2.412, 4.011) | < 0.001 | 1.283 (0.875, 1.880) | 0.201 | 1.053 (0.675, 1.642) | 0.818 | 1.035 (0.662, 1.616) | 0.880 |
| SAD/height*^β^* ≥ 14.649 | 4.190 (3.249, 5.422) | < 0.001 | 2.432 (1.681, 3.532) | < 0.001 | 2.476 (1.623, 3.792) | < 0.001 | 2.453 (1.606, 3.761) | < 0.001 |

BMI, body mass index; VAT, visceral adipose tissue area; SAT, subcutaneous adipose tissue area; TAT, total abdominal adipose tissue area; WC, waist circumference; TAD, transverse diameter; SAD, sagittal diameter; OR, odds ratio; 95% CI, 95% confidence interval.

^1^ Adjustment for age and BMI.

^2^ Adjustment for age and WC.

^3^ Adjustment for age, BMI and WC.

**Table S14 Association of Height-Normalized Body Composition Indicators with C**ancers

|  | Crude OR (95% CI) | *P* value | Adj. OR (95% CI)^1^ | *P* value | Adj. OR (95% CI)^2^ | *P* value | Adj. OR (95% CI)^3^ | *P* value |
| --- | --- | --- | --- | --- | --- | --- | --- | --- |
| Male |  |  |  |  |  |  |  |  |
| BMI ≥ 24 | 1.104 (0.853, 1.431) | 0.453 | - | - | 0.744 (0.508, 1.090) | 0.129 | - | - |
| VAT/height*^β^* ≥ 40.088 | 1.898 (1.442, 2.510) | < 0.001 | 1.704 (1.164, 2.509) | 0.006 | 1.282 (0.862, 1.910) | 0.222 | 1.397 (0.933, 2.100) | 0.106 |
| SAT/height*^β^* ≥ 45.633 | 1.206 (0.852, 1.720) | 0.295 | 1.575 (1.003, 2.497) | 0.051 | 1.243 (0.792, 1.968) | 0.349 | 1.380 (0.872, 2.205) | 0.173 |
| TAT/height*^β^* ≥ 68.293 | 1.533 (1.190, 1.981) | 0.001 | 1.564 (1.074, 2.286) | 0.020 | 1.126 (0.756, 1.676) | 0.559 | 1.236 (0.823, 1.858) | 0.307 |
| WC/height*^β^* ≥ 63.790 | 1.915 (1.482, 2.483) | < 0.001 | 2.187 (1.481, 3.251) | < 0.001 | - | - | - | - |
| TAD/height*^β^* ≥ 22.645 | 1.747 (1.333, 2.298) | < 0.001 | 1.784 (1.203, 2.662) | 0.004 | 1.281 (0.835, 1.969) | 0.258 | 1.379 (0.894, 2.135) | 0.148 |
| SAD/height*^β^* ≥ 15.233 | 2.215 (1.712, 2.877) | < 0.001 | 2.693 (1.877, 3.890) | < 0.001 | 2.350 (1.564, 3.549) | < 0.001 | 2.340 (1.554, 3.540) | < 0.001 |
| Female |  |  |  |  |  |  |  |  |
| BMI ≥ 24 | 2.249 (1.713, 2.959) | < 0.001 | - | - | 1.814 (1.230, 2.684) | 0.003 | - | - |
| VAT/height*^β^* ≥ 37.894 | 2.967 (2.324, 3.799) | < 0.001 | 1.438 (1.034, 1.999) | 0.031 | 1.558 (1.107, 2.193) | 0.011 | 1.495 (1.057, 2.114) | 0.023 |
| SAT/height*^β^* ≥ 70.819 | 1.927 (1.510, 2.462) | < 0.001 | 1.195 (0.850, 1.679) | 0.305 | 1.300 (0.900, 1.877) | 0.162 | 1.241 (0.856, 1.800) | 0.254 |
| TAT/height*^β^* ≥ 110.015 | 2.355 (1.847, 3.008) | < 0.001 | 1.191 (0.837, 1.695) | 0.331 | 1.316 (0.892, 1.942) | 0.167 | 1.250 (0.843, 1.855) | 0.267 |
| WC/height*^β^* ≥ 62.301 | 2.357 (1.852, 3.007) | < 0.001 | 1.341 (0.950, 1.894) | 0.095 | - | - | - | - |
| TAD/height*^β^* ≥ 21.703 | 2.087 (1.640, 2.660) | < 0.001 | 1.056 (0.752, 1.480) | 0.754 | 1.096 (0.735, 1.632) | 0.653 | 1.085 (0.727, 1.619) | 0.688 |
| SAD/height*^β^* ≥ 14.684 | 2.764 (2.152, 3.558) | < 0.001 | 1.815 (1.286, 2.568) | < 0.001 | 2.195 (1.491, 3.242) | < 0.001 | 2.137 (1.450, 3.161) | < 0.001 |

BMI, body mass index; VAT, visceral adipose tissue area; SAT, subcutaneous adipose tissue area; TAT, total abdominal adipose tissue area; WC, waist circumference; TAD, transverse diameter; SAD, sagittal diameter; OR, odds ratio; 95% CI, 95% confidence interval.

^1^ Adjustment for age and BMI.

^2^ Adjustment for age and WC.

^3^ Adjustment for age, BMI and WC.

**Table S15 Association of Height-Normalized Body Composition Indicators with A**bnormal **B**one **M**ass

|  | Crude OR (95% CI) | *P* value | Adj. OR (95% CI)^1^ | *P* value | Adj. OR (95% CI)^2^ | *P* value | Adj. OR (95% CI)^3^ | *P* value |
| --- | --- | --- | --- | --- | --- | --- | --- | --- |
| Male |  |  |  |  |  |  |  |  |
| BMI ≥ 24 | 0.755 (0.501, 1.122) | 0.17 | - | - | 0.358 (0.175, 0.713) | 0.004 | - | - |
| VAT/height*^β^* ≥ 32.740 | 2.762 (1.891, 4.062) | < 0.001 | 2.603 (1.428, 4.812) | 0.002 | 1.511 (0.779, 2.947) | 0.223 | 1.508 (0.769, 2.975) | 0.233 |
| SAT/height*^β^* ≥ 22.830 | 1.516 (0.972, 2.419) | 0.073 | 1.137 (0.571, 2.295) | 0.716 | 0.577 (0.276, 1.206) | 0.142 | 0.624 (0.296, 1.320) | 0.215 |
| TAT/height*^β^* ≥ 57.282 | 2.224 (1.500, 3.341) | < 0.001 | 2.247 (1.189, 4.320) | 0.014 | 1.171 (0.583, 2.368) | 0.659 | 1.221 (0.600, 2.505) | 0.583 |
| WC/height*^β^* ≥ 62.677 | 2.350 (1.612, 3.447) | < 0.001 | 3.611 (1.849, 7.225) | < 0.001 | - | - | - | - |
| TAD/height*^β^* ≥ 21.894 | 2.159 (1.484, 3.160) | < 0.001 | 3.661 (1.856, 7.408) | < 0.001 | 1.704 (0.821, 3.571) | 0.154 | 2.126 (0.997, 4.603) | 0.053 |
| SAD/height*^β^* ≥ 14.470 | 2.465 (1.669, 3.687) | < 0.001 | 2.438 (1.288, 4.695) | 0.007 | 1.268 (0.635, 2.548) | 0.502 | 1.393 (0.688, 2.842) | 0.359 |
| Female |  |  |  |  |  |  |  |  |
| BMI ≥ 24 | 1.972 (1.463, 2.658) | < 0.001 | - | - | 0.954 (0.541, 1.679) | 0.871 | - | - |
| VAT/height*^β^* ≥ 44.030 | 7.254 (5.440, 9.740) | < 0.001 | 2.873 (1.710, 4.871) | < 0.001 | 2.434 (1.416, 4.212) | 0.001 | 2.650 (1.528, 4.629) | < 0.001 |
| SAT/height*^β^* ≥ 69.707 | 2.185 (1.678, 2.849) | < 0.001 | 0.962 (0.579, 1.590) | 0.879 | 0.688 (0.392, 1.198) | 0.189 | 0.713 (0.405, 1.247) | 0.238 |
| TAT/height*^β^* ≥ 108.829 | 4.651 (3.523, 6.177) | < 0.001 | 2.370 (1.379, 4.112) | 0.002 | 1.968 (1.073, 3.629) | 0.029 | 2.110 (1.144, 3.914) | 0.017 |
| WC/height*^β^* ≥ 63.899 | 4.233 (3.221, 5.585) | < 0.001 | 1.789 (1.048, 3.069) | 0.034 | - | - | - | - |
| TAD/height*^β^* ≥ 22.280 | 3.712 (2.831, 4.887) | < 0.001 | 1.574 (0.924, 2.689) | 0.095 | 1.175 (0.637, 2.169) | 0.605 | 1.237 (0.668, 2.291) | 0.498 |
| SAD/height*^β^* ≥ 14.856 | 4.165 (3.160, 5.509) | < 0.001 | 1.863 (1.097, 3.184) | 0.022 | 1.483 (0.818, 2.700) | 0.195 | 1.561 (0.857, 2.854) | 0.146 |

BMI, body mass index; VAT, visceral adipose tissue area; SAT, subcutaneous adipose tissue area; TAT, total abdominal adipose tissue area; WC, waist circumference; TAD, transverse diameter; SAD, sagittal diameter; OR, odds ratio; 95% CI, 95% confidence interval.

^1^ Adjustment for age and BMI.

^2^ Adjustment for age and WC.

^3^ Adjustment for age, BMI and WC.

**Table S16 Patient Distribution by Number and Combination of Comorbidities**

| **Conditions** | **Diseases** | **N** | **Conditions** | **Diseases** | **N** |
| --- | --- | --- | --- | --- | --- |
| **Single Comorbidity** | Dyslipidemia | 718 | **Triple Comorbidities** | Dyslipidemia + Type 2 diabetes + Cardio-cerebrovascular diseases | 45 |
|  | Type 2 diabetes | 239 |  | Dyslipidemia + Type 2 diabetes + Cancers | 0 |
|  | Cardio-cerebrovascular diseases | 494 |  | Dyslipidemia + Type 2 diabetes + Abnormal bone mass | 31 |
|  | Cancers | 738 |  | Dyslipidemia + Cardio-cerebrovascular diseases + Cancers | 0 |
|  | Abnormal bone mass | 210 |  | Dyslipidemia + Cardio-cerebrovascular diseases + Abnormal bone mass | 9 |
| **Dual Comorbidities** | Dyslipidemia + Type 2 diabetes | 177 |  | Dyslipidemia + Cancers + Abnormal bone mass | 0 |
|  | Dyslipidemia + Cardio-cerebrovascular diseases | 95 |  | Type 2 diabetes + Cardio-cerebrovascular diseases + Cancers | 0 |
|  | Dyslipidemia + Cancers | 0 |  | Type 2 diabetes + Cardio-cerebrovascular diseases + Abnormal bone mass | 22 |
|  | Dyslipidemia + Abnormal bone mass | 82 |  | Type 2 diabetes + Cancers + Abnormal bone mass | 0 |
|  | Type 2 diabetes + Cardio-cerebrovascular diseases | 261 |  | Cardio-cerebrovascular diseases + Cancers + Abnormal bone mass | 20 |
|  | Type 2 diabetes + Cancers | 0 | **Quadruple Comorbidities** | Dyslipidemia + Type 2 diabetes + Cardio-cerebrovascular diseases + Cancers | 0 |
|  | Type 2 diabetes + Abnormal bone mass | 26 |  | Dyslipidemia + Type 2 diabetes + Cardio-cerebrovascular diseases + Abnormal bone mass | 2 |
|  | Cardio-cerebrovascular diseases + Cancers | 292 |  | Dyslipidemia + Type 2 diabetes + Cancers + Abnormal bone mass | 0 |
|  | Cardio-cerebrovascular diseases + Abnormal bone mass | 49 |  | Dyslipidemia + Cardio-cerebrovascular diseases + Cancers + Abnormal bone mass | 0 |
|  | Cancers + Abnormal bone mass | 58 |  | Type 2 diabetes + Cardio-cerebrovascular diseases + Cancers + Abnormal bone mass | 0 |
|  |  |  | **Quintuple Comorbidities** | Dyslipidemia + Type 2 diabetes + Cardio-cerebrovascular diseases + Cancers + Abnormal bone mass | 0 |

**Table S17 Patient Distribution by Number of Comorbidities**

| Diseases | Single Comorbidity | Dual Comorbidities | Triple Comorbidities | Quadruple Comorbidities | Quintuple Comorbidities | Total |
| --- | --- | --- | --- | --- | --- | --- |
| Dyslipidemia | 718 | 354 | 85 | 2 | 0 | 1159 |
| Type 2 diabetes | 239 | 464 | 98 | 2 | 0 | 803 |
| Cardio-cerebrovascular diseases | 494 | 697 | 96 | 2 | 0 | 1289 |
| Cancers | 738 | 350 | 20 | 0 | 0 | 1108 |
| Abnormal bone mass | 210 | 215 | 82 | 2 | 0 | 509 |

**Table S18 Association of Height-Normalized Body Composition Indicators with Dyslipidemia (N=718)**

|  | Crude OR (95% CI) | *P* value | Adj. OR (95% CI)^1^ | *P* value | Adj. OR (95% CI)^2^ | *P* value | Adj. OR (95% CI)^3^ | *P* value |
| --- | --- | --- | --- | --- | --- | --- | --- | --- |
| Male |  |  |  |  |  |  |  |  |
| BMI ≥ 24 | 3.135 (2.382, 4.140) | < 0.001 | - | - | 1.707 (1.169, 2.497) | 0.006 | - | - |
| VAT/height*^β^* ≥ 34.224 | 4.153 (3.138, 5.520) | < 0.001 | 2.969 (2.069, 4.283) | < 0.001 | 3.039 (2.063, 4.501) | < 0.001 | 2.954 (2.002, 4.383) | < 0.001 |
| SAT/height*^β^* ≥ 31.642 | 2.652 (2.013, 3.505) | < 0.001 | 1.412 (1.001, 1.993) | 0.049 | 1.340 (0.936, 1.917) | 0.110 | 1.281 (0.891, 1.838) | 0.180 |
| TAT/height*^β^* ≥ 66.692 | 4.652 (3.500, 6.217) | < 0.001 | 3.501 (2.394, 5.154) | < 0.001 | 3.771 (2.489, 5.751) | < 0.001 | 3.655 (2.406, 5.586) | < 0.001 |
| WC/height*^β^* ≥ 63.194 | 3.809 (2.883, 5.054) | < 0.001 | 2.470 (1.674, 3.657) | < 0.001 | - | - | - | - |
| TAD/height*^β^* ≥ 22.080 | 3.326 (2.525, 4.397) | < 0.001 | 1.923 (1.316, 2.817) | < 0.001 | 1.856 (1.224, 2.821) | 0.004 | 1.769 (1.162, 2.698) | 0.008 |
| SAD/height*^β^* ≥ 15.228 | 3.605 (2.731, 4.779) | < 0.001 | 2.237 (1.562, 3.212) | < 0.001 | 2.192 (1.464, 3.293) | < 0.001 | 2.183 (1.456, 3.285) | < 0.001 |
| Female |  |  |  |  |  |  |  |  |
| BMI ≥ 24 | 3.128 (2.275, 4.305) | < 0.001 | - | - | 1.509 (0.979, 2.325) | 0.062 | - | - |
| VAT/height*^β^* ≥ 44.095 | 4.657 (3.424, 6.367) | < 0.001 | 3.182 (2.139, 4.757) | < 0.001 | 3.389 (2.254, 5.126) | < 0.001 | 3.183 (2.108, 4.835) | < 0.001 |
| SAT/height*^β^* ≥ 73.172 | 2.959 (2.192, 4.006) | < 0.001 | 1.493 (1.014, 2.194) | 0.042 | 1.451 (0.956, 2.203) | 0.080 | 1.395 (0.914, 2.127) | 0.122 |
| TAT/height*^β^* ≥ 127.606 | 3.924 (2.876, 5.368) | < 0.001 | 2.153 (1.411, 3.290) | < 0.001 | 2.285 (1.450, 3.613) | < 0.001 | 2.126 (1.342, 3.378) | 0.001 |
| WC/height*^β^* ≥ 64.509 | 3.890 (2.863, 5.302) | < 0.001 | 2.151 (1.398, 3.313) | < 0.001 | - | - | - | - |
| TAD/height*^β^* ≥ 22.852 | 3.283 (2.419, 4.466) | < 0.001 | 1.572 (1.030, 2.395) | 0.036 | 1.559 (0.978, 2.489) | 0.062 | 1.462 (0.911, 2.347) | 0.115 |
| SAD/height*^β^* ≥ 15.341 | 4.391 (3.176, 6.088) | < 0.001 | 2.500 (1.646, 3.803) | < 0.001 | 2.663 (1.698, 4.195) | < 0.001 | 2.532 (1.608, 4.006) | < 0.001 |

BMI, body mass index; VAT, visceral adipose tissue area; SAT, subcutaneous adipose tissue area; TAT, total abdominal adipose tissue area; WC, waist circumference; TAD, transverse diameter; SAD, sagittal diameter; OR, odds ratio; 95% CI, 95% confidence interval.

^1^ Adjustment for age and BMI.

^2^ Adjustment for age and WC.

^3^ Adjustment for age, BMI and WC.

**Table S19 Association of Height-Normalized Body Composition Indicators with Type 2 Diabetes (N=239)**

|  | Crude OR (95% CI) | *P* value | Adj. OR (95% CI)^1^ | *P* value | Adj. OR (95% CI)^2^ | *P* value | Adj. OR (95% CI)^3^ | *P* value |
| --- | --- | --- | --- | --- | --- | --- | --- | --- |
| Male |  |  |  |  |  |  |  |  |
| BMI ≥ 24 | 1.855 (1.252, 2.752) | 0.002 | - | - | 0.945 (0.542, 1.638) | 0.841 | - | - |
| VAT/height*^β^* ≥ 34.824 | 2.930 (1.969, 4.389) | < 0.001 | 2.095 (1.261, 3.502) | 0.004 | 1.690 (0.974, 2.945) | 0.063 | 1.705 (0.981, 2.972) | 0.059 |
| SAT/height*^β^* ≥ 23.272 | 1.472 (0.931, 2.386) | 0.106 | 0.789 (0.447, 1.408) | 0.417 | 0.603 (0.332, 1.101) | 0.097 | 0.608 (0.334, 1.112) | 0.104 |
| TAT/height*^β^* ≥ 66.728 | 2.321 (1.565, 3.462) | < 0.001 | 1.520 (0.908, 2.554) | 0.112 | 1.123 (0.633, 1.990) | 0.692 | 1.130 (0.636, 2.005) | 0.675 |
| WC/height*^β^* ≥ 63.088 | 2.468 (1.662, 3.689) | < 0.001 | 1.631 (0.963, 2.779) | 0.070 | - | - | - | - |
| Female |  |  |  |  |  |  |  |  |
| BMI ≥ 24 | 3.812 (2.470, 5.878) | < 0.001 | - | - | 0.920 (0.492, 1.700) | 0.791 | - | - |
| VAT/height*^β^* ≥ 47.488 | 6.964 (4.471, 11.050) | < 0.001 | 2.829 (1.586, 5.092) | < 0.001 | 1.942 (1.057, 3.582) | 0.033 | 2.018 (1.091, 3.748) | 0.025 |
| SAT/height*^β^* ≥ 72.682 | 4.359 (2.836, 6.795) | < 0.001 | 2.021 (1.160, 3.539) | 0.013 | 1.185 (0.630, 2.223) | 0.597 | 1.189 (0.632, 2.232) | 0.590 |
| TAT/height*^β^* ≥ 113.968 | 6.714 (4.248, 10.906) | < 0.001 | 3.004 (1.644, 5.559) | < 0.001 | 1.845 (0.940, 3.646) | 0.076 | 1.873 (0.953, 3.709) | 0.070 |
| WC/height*^β^* ≥ 64.652 | 6.177 (3.994, 9.685) | < 0.001 | 2.657 (1.447, 4.908) | 0.002 | - | - | - | - |

The AUC values of 1 for both TAD (TAD/height*^β^*) and SAD (SAD/height*^β^*) in both genders suggest flawless distinction between different samples across all thresholds. In such cases, logistic regression models for binary classification can't yield meaningful coefficient estimates or odds ratios due to the perfect discrimination. BMI, body mass index; VAT, visceral adipose tissue area; SAT, subcutaneous adipose tissue area; TAT, total abdominal adipose tissue area; WC, waist circumference; OR, odds ratio; 95% CI, 95% confidence interval.

^1^ Adjustment for age and BMI.

^2^ Adjustment for age and WC.

^3^ Adjustment for age, BMI and WC.

**Table S20 Association of Height-Normalized Body Composition Indicators with Cardio-Cerebrovascular Diseases (N=494)**

|  | Crude OR (95% CI) | *P* value | Adj. OR (95% CI)^1^ | *P* value | Adj. OR (95% CI)^2^ | *P* value | Adj. OR (95% CI)^3^ | *P* value |
| --- | --- | --- | --- | --- | --- | --- | --- | --- |
| Male |  |  |  |  |  |  |  |  |
| BMI ≥ 24 | 1.770 (1.312, 2.392) | < 0.001 | - | - | 0.917 (0.593, 1.415) | 0.696 | - | - |
| VAT/height*^β^* ≥ 36.959 | 3.135 (2.305, 4.281) | < 0.001 | 2.683 (1.792, 4.044) | < 0.001 | 2.087 (1.347, 3.249) | 0.001 | 2.111 (1.360, 3.290) | < 0.001 |
| SAT/height*^β^* ≥ 45.681 | 2.056 (1.410, 3.014) | < 0.001 | 1.870 (1.155, 3.048) | 0.011 | 1.444 (0.890, 2.355) | 0.138 | 1.585 (0.968, 2.610) | 0.068 |
| TAT/height*^β^* ≥ 68.798 | 2.459 (1.821, 3.332) | < 0.001 | 2.074 (1.369, 3.157) | < 0.001 | 1.477 (0.939, 2.327) | 0.091 | 1.530 (0.970, 2.417) | 0.067 |
| WC/height*^β^* ≥ 63.842 | 2.721 (2.011, 3.696) | < 0.001 | 2.453 (1.582, 3.824) | < 0.001 | - | - | - | - |
| TAD/height*^β^* ≥ 22.645 | 2.476 (1.815, 3.390) | < 0.001 | 2.236 (1.437, 3.499) | < 0.001 | 1.544 (0.954, 2.505) | 0.077 | 1.625 (1.000, 2.648) | 0.051 |
| SAD/height*^β^* ≥ 15.319 | 3.358 (2.473, 4.581) | < 0.001 | 3.486 (2.290, 5.352) | < 0.001 | 2.805 (1.762, 4.497) | < 0.001 | 2.824 (1.772, 4.535) | < 0.001 |
| Female |  |  |  |  |  |  |  |  |
| BMI ≥ 24 | 2.959 (2.071, 4.223) | < 0.001 | - | - | 1.798 (1.064, 3.042) | 0.028 | - | - |
| VAT/height*^β^* ≥ 37.717 | 3.916 (2.765, 5.603) | < 0.001 | 1.787 (1.123, 2.851) | 0.015 | 1.675 (1.035, 2.721) | 0.036 | 1.682 (1.036, 2.740) | 0.036 |
| SAT/height*^β^* ≥ 79.154 | 2.457 (1.722, 3.497) | < 0.001 | 1.410 (0.853, 2.323) | 0.178 | 1.264 (0.741, 2.151) | 0.388 | 1.265 (0.738, 2.162) | 0.390 |
| TAT/height*^β^* ≥ 110.633 | 2.855 (2.040, 4.014) | < 0.001 | 1.286 (0.775, 2.126) | 0.328 | 1.109 (0.636, 1.931) | 0.715 | 1.107 (0.632, 1.936) | 0.721 |
| WC/height*^β^* ≥ 65.280 | 3.213 (2.264, 4.560) | < 0.001 | 1.653 (0.972, 2.814) | 0.063 | - | - | - | - |
| TAD/height*^β^* ≥ 22.688 | 2.741 (1.950, 3.856) | < 0.001 | 1.412 (0.851, 2.335) | 0.179 | 1.227 (0.688, 2.184) | 0.488 | 1.226 (0.686, 2.186) | 0.490 |
| SAD/height*^β^* ≥ 14.649 | 2.942 (2.096, 4.137) | < 0.001 | 1.927 (1.172, 3.173) | 0.010 | 1.816 (1.053, 3.143) | 0.032 | 1.827 (1.055, 3.173) | 0.032 |

BMI, body mass index; VAT, visceral adipose tissue area; SAT, subcutaneous adipose tissue area; TAT, total abdominal adipose tissue area; WC, waist circumference; TAD, transverse diameter; SAD, sagittal diameter; OR, odds ratio; 95% CI, 95% confidence interval.

^1^ Adjustment for age and BMI.

^2^ Adjustment for age and WC.

^3^ Adjustment for age, BMI and WC.

**Table S21 Association of Height-Normalized Body Composition Indicators with Cancers (N=738)**

|  | Crude OR (95% CI) | *P* value | Adj. OR (95% CI)^1^ | *P* value | Adj. OR (95% CI)^2^ | *P* value | Adj. OR (95% CI)^3^ | *P* value |
| --- | --- | --- | --- | --- | --- | --- | --- | --- |
| Male |  |  |  |  |  |  |  |  |
| BMI ≥ 24 | 1.201 (0.902, 1.598) | 0.210 | - | - | 0.714 (0.470, 1.083) | 0.114 | - | - |
| VAT/height*^β^* ≥ 40.088 | 2.169 (1.607, 2.938) | < 0.001 | 1.872 (1.239, 2.846) | 0.003 | 1.384 (0.903, 2.125) | 0.137 | 1.524 (0.985, 2.365) | 0.060 |
| SAT/height*^β^* ≥ 45.633 | 1.376 (0.944, 2.015) | 0.099 | 1.661 (1.023, 2.717) | 0.042 | 1.288 (0.791, 2.109) | 0.311 | 1.420 (0.865, 2.347) | 0.168 |
| TAT/height*^β^* ≥ 68.293 | 1.699 (1.283, 2.254) | < 0.001 | 1.499 (1.002, 2.250) | 0.050 | 1.052 (0.684, 1.617) | 0.817 | 1.137 (0.733, 1.763) | 0.567 |
| WC/height*^β^* ≥ 63.790 | 2.162 (1.628, 2.879) | < 0.001 | 2.187 (1.444, 3.335) | < 0.001 | - | - | - | - |
| TAD/height*^β^* ≥ 22.645 | 1.856 (1.380, 2.505) | < 0.001 | 1.803 (1.178, 2.776) | 0.007 | 1.254 (0.790, 1.994) | 0.337 | 1.352 (0.846, 2.166) | 0.208 |
| SAD/height*^β^* ≥ 15.233 | 2.553 (1.920, 3.406) | < 0.001 | 2.829 (1.923, 4.195) | < 0.001 | 2.463 (1.580, 3.862) | < 0.001 | 2.415 (1.546, 3.791) | < 0.001 |
| Female |  |  |  |  |  |  |  |  |
| BMI ≥ 24 | 2.196 (1.630, 2.961) | < 0.001 | - | - | 1.948 (1.285, 2.960) | 0.002 | - | - |
| VAT/height*^β^* ≥ 37.894 | 2.817 (2.152, 3.698) | < 0.001 | 1.478 (1.034, 2.114) | 0.032 | 1.677 (1.154, 2.441) | 0.007 | 1.602 (1.098, 2.341) | 0.015 |
| SAT/height*^β^* ≥ 70.819 | 2.096 (1.603, 2.744) | < 0.001 | 1.397 (0.967, 2.018) | 0.075 | 1.643 (1.105, 2.448) | 0.014 | 1.560 (1.045, 2.335) | 0.030 |
| TAT/height*^β^* ≥ 110.015 | 2.384 (1.824, 3.123) | < 0.001 | 1.329 (0.906, 1.949) | 0.145 | 1.598 (1.049, 2.442) | 0.030 | 1.510 (0.985, 2.319) | 0.059 |
| WC/height*^β^* ≥ 62.301 | 2.215 (1.698, 2.897) | < 0.001 | 1.314 (0.903, 1.914) | 0.153 | - | - | - | - |
| TAD/height*^β^* ≥ 21.703 | 1.887 (1.447, 2.465) | < 0.001 | 0.963 (0.664, 1.393) | 0.840 | 1.040 (0.672, 1.607) | 0.860 | 1.022 (0.659, 1.582) | 0.922 |
| SAD/height*^β^* ≥ 14.684 | 2.530 (1.922, 3.335) | < 0.001 | 1.762 (1.216, 2.559) | 0.003 | 2.236 (1.474, 3.406) | < 0.001 | 2.169 (1.428, 3.308) | < 0.001 |

BMI, body mass index; VAT, visceral adipose tissue area; SAT, subcutaneous adipose tissue area; TAT, total abdominal adipose tissue area; WC, waist circumference; TAD, transverse diameter; SAD, sagittal diameter; OR, odds ratio; 95% CI, 95% confidence interval.

^1^ Adjustment for age and BMI.

^2^ Adjustment for age and WC.

^3^ Adjustment for age, BMI and WC.

**Table S22 Association of Height-Normalized Body Composition Indicators with Abnormal Bone Mass (N=210)**

|  | Crude OR (95% CI) | *P* value | Adj. OR (95% CI)^1^ | *P* value | Adj. OR (95% CI)^2^ | *P* value | Adj. OR (95% CI)^3^ | *P* value |
| --- | --- | --- | --- | --- | --- | --- | --- | --- |
| Male |  |  |  |  |  |  |  |  |
| BMI ≥ 24 | 0.583 (0.306, 1.056) | 0.086 | - | - | 0.518 (0.176, 1.444) | 0.218 | - | - |
| VAT/height*^β^* ≥ 32.740 | 2.375 (1.386, 4.122) | 0.002 | 2.051 (0.794, 5.439) | 0.141 | 1.269 (0.456, 3.567) | 0.648 | 1.386 (0.491, 3.967) | 0.538 |
| SAT/height*^β^* ≥ 22.830 | 1.054 (0.584, 1.990) | 0.865 | 0.838 (0.295, 2.446) | 0.742 | 0.446 (0.145, 1.377) | 0.156 | 0.495 (0.159, 1.553) | 0.223 |
| TAT/height*^β^* ≥ 57.282 | 2.013 (1.150, 3.644) | 0.017 | 2.244 (0.828, 6.415) | 0.120 | 1.326 (0.451, 4.017) | 0.611 | 1.490 (0.497, 4.629) | 0.481 |
| WC/height*^β^* ≥ 62.677 | 1.821 (1.065, 3.137) | 0.029 | 2.278 (0.825, 6.512) | 0.116 | - | - | - | - |
| TAD/height*^β^* ≥ 21.894 | 1.886 (1.103, 3.257) | 0.021 | 2.791 (0.979, 8.338) | 0.059 | 1.459 (0.468, 4.606) | 0.515 | 1.860 (0.578, 6.120) | 0.300 |
| SAD/height*^β^* ≥ 14.470 | 2.205 (1.267, 3.956) | 0.006 | 2.610 (0.953, 7.561) | 0.068 | 1.515 (0.529, 4.488) | 0.443 | 1.819 (0.613, 5.642) | 0.288 |
| Female |  |  |  |  |  |  |  |  |
| BMI ≥ 24 | 1.869 (1.243, 2.782) | 0.002 | - | - | 1.189 (0.569, 2.470) | 0.643 | - | - |
| VAT/height*^β^* ≥ 44.030 | 8.007 (5.370, 12.169) | < 0.001 | 3.244 (1.650, 6.512) | < 0.001 | 3.139 (1.557, 6.435) | 0.002 | 3.323 (1.626, 6.918) | 0.001 |
| SAT/height*^β^* ≥ 69.707 | 1.941 (1.352, 2.787) | < 0.001 | 0.912 (0.473, 1.746) | 0.782 | 0.767 (0.375, 1.546) | 0.461 | 0.774 (0.376, 1.572) | 0.481 |
| TAT/height*^β^* ≥ 108.829 | 5.030 (3.413, 7.535) | < 0.001 | 2.355 (1.172, 4.807) | 0.017 | 2.389 (1.088, 5.333) | 0.031 | 2.469 (1.116, 5.554) | 0.027 |
| WC/height*^β^* ≥ 63.899 | 3.919 (2.710, 5.704) | < 0.001 | 1.344 (0.680, 2.661) | 0.394 | - | - | - | - |
| TAD/height*^β^* ≥ 22.280 | 3.233 (2.240, 4.702) | < 0.001 | 1.058 (0.529, 2.107) | 0.872 | 0.854 (0.386, 1.878) | 0.694 | 0.865 (0.388, 1.917) | 0.721 |
| SAD/height*^β^* ≥ 14.856 | 4.056 (2.801, 5.899) | < 0.001 | 1.700 (0.860, 3.370) | 0.126 | 1.595 (0.739, 3.462) | 0.235 | 1.625 (0.750, 3.544) | 0.219 |

BMI, body mass index; VAT, visceral adipose tissue area; SAT, subcutaneous adipose tissue area; TAT, total abdominal adipose tissue area; WC, waist circumference; TAD, transverse diameter; SAD, sagittal diameter; OR, odds ratio; 95% CI, 95% confidence interval.

^1^ Adjustment for age and BMI.

^2^ Adjustment for age and WC.

^3^ Adjustment for age, BMI and WC.

**Table S23 Association of Unnormalized Body Composition Indicators with Dyslipidemia**

|  | Crude OR (95% CI) | *P* value | Adj. OR (95% CI)^1^ | *P* value | Adj. OR (95% CI)^2^ | *P* value | Adj. OR (95% CI)^3^ | *P* value |
| --- | --- | --- | --- | --- | --- | --- | --- | --- |
| Male |  |  |  |  |  |  |  |  |
| Weight ≥ 68.2 | 2.716 (2.124, 3.481) | < 0.001 | 3.331 (2.548, 4.374) | < 0.001 | 1.132 (0.798, 1.604) | 0.485 | 1.363 (0.945, 1.966) | 0.097 |
| VAT ≥ 112.026 | 4.619 (3.576, 5.993) | < 0.001 | 3.202 (2.311, 4.455) | < 0.001 | 2.988 (2.091, 4.287) | < 0.001 | 2.977 (2.082, 4.272) | < 0.001 |
| SAT ≥ 109.970 | 2.664 (2.083, 3.414) | < 0.001 | 1.393 (1.018, 1.905) | 0.038 | 1.180 (0.845, 1.645) | 0.330 | 1.157 (0.828, 1.615) | 0.392 |
| TAT ≥ 227.084 | 4.514 (3.501, 5.843) | < 0.001 | 3.103 (2.211, 4.372) | < 0.001 | 2.893 (1.976, 4.249) | < 0.001 | 2.878 (1.965, 4.229) | < 0.001 |
| WC ≥ 86.952 | 4.266 (3.309, 5.519) | < 0.001 | 2.785 (1.961, 3.967) | < 0.001 | - | - | - | - |
| TAD ≥ 31.047 | 3.752 (2.907, 4.866) | < 0.001 | 2.218 (1.579, 3.127) | < 0.001 | 1.884 (1.282, 2.773) | 0.001 | 1.872 (1.274, 2.757) | 0.001 |
| SAD ≥ 20.960 | 3.865 (3.004, 4.993) | < 0.001 | 2.396 (1.727, 3.333) | < 0.001 | 2.072 (1.419, 3.032) | < 0.001 | 2.105 (1.440, 3.084) | < 0.001 |
| Female |  |  |  |  |  |  |  |  |
| Weight ≥ 57.01 | 2.613 (2.023, 3.382) | < 0.001 | 2.901 (2.198, 3.844) | < 0.001 | 1.133 (0.797, 1.609) | 0.485 | 1.345 (0.932, 1.941) | 0.113 |
| VAT ≥ 77.414 | 5.354 (4.088, 7.043) | < 0.001 | 3.282 (2.323, 4.655) | < 0.001 | 3.540 (2.461, 5.119) | < 0.001 | 3.371 (2.337, 4.886) | < 0.001 |
| SAT ≥ 152.005 | 2.597 (2.011, 3.361) | < 0.001 | 1.087 (0.778, 1.515) | 0.622 | 0.923 (0.633, 1.342) | 0.675 | 0.924 (0.632, 1.347) | 0.681 |
| TAT ≥ 244.269 | 3.982 (3.055, 5.210) | < 0.001 | 1.887 (1.313, 2.712) | < 0.001 | 1.938 (1.295, 2.905) | 0.001 | 1.839 (1.224, 2.768) | 0.003 |
| WC ≥ 87.250 | 4.377 (3.308, 5.817) | < 0.001 | 2.051 (1.395, 3.020) | < 0.001 | - | - | - | - |
| TAD ≥ 31.291 | 3.957 (2.968, 5.298) | < 0.001 | 1.625 (1.101, 2.401) | 0.015 | 1.588 (1.026, 2.463) | 0.038 | 1.502 (0.966, 2.338) | 0.071 |
| SAD ≥ 19.130 | 3.712 (2.858, 4.838) | < 0.001 | 1.801 (1.273, 2.550) | < 0.001 | 1.798 (1.210, 2.674) | 0.004 | 1.766 (1.185, 2.635) | 0.005 |

BMI, body mass index; VAT, visceral adipose tissue area; SAT, subcutaneous adipose tissue area; TAT, total abdominal adipose tissue area; WC, waist circumference; TAD, transverse diameter; SAD, sagittal diameter; OR, odds ratio; 95% CI, 95% confidence interval.

^1^ Adjustment for age and BMI. Weight was adjusted for age and height.

^2^ Adjustment for age and WC.

^3^ Adjustment for age, BMI and WC. Weight was adjusted for age, height and WC.

**Table S24 Association of Unnormalized Body Composition Indicators with Type 2 Diabetes**

|  | Crude OR (95% CI) | *P* value | Adj. OR (95% CI)^1^ | *P* value | Adj. OR (95% CI)^2^ | *P* value | Adj. OR (95% CI)^3^ | *P* value |
| --- | --- | --- | --- | --- | --- | --- | --- | --- |
| Male |  |  |  |  |  |  |  |  |
| Weight ≥ 68.45 | 1.933 (1.487, 2.519) | < 0.001 | 2.930 (2.152, 4.016) | < 0.001 | 0.743 (0.497, 1.108) | 0.146 | 0.922 (0.645, 1.402) | 0.703 |
| VAT ≥ 112.351 | 4.790 (3.630, 6.351) | < 0.001 | 3.518 (2.459, 5.062) | < 0.001 | 2.458 (1.650, 3.674) | < 0.001 | 2.456 (1.649, 3.672) | < 0.001 |
| SAT ≥ 97.125 | 1.718 (1.312, 2.253) | < 0.001 | 0.824 (0.572, 1.181) | 0.294 | 0.521 (0.352, 0.765) | < 0.001 | 0.538 (0.363, 0.793) | 0.002 |
| TAT ≥ 227.392 | 3.599 (2.745, 4.737) | < 0.001 | 2.345 (1.621, 3.404) | < 0.001 | 1.393 (0.917, 2.117) | 0.12 | 1.419 (0.933, 2.158) | 0.102 |
| WC ≥ 87.948 | 4.092 (3.111, 5.404) | < 0.001 | 2.988 (2.040, 4.398) | < 0.001 | - | - | - | - |
| Female |  |  |  |  |  |  |  |  |
| Weight ≥ 57.55 | 2.899 (2.180, 3.869) | < 0.001 | 3.081 (2.201, 4.338) | < 0.001 | 1.052 (0.693, 1.595) | 0.811 | 1.230 (0.797, 1.896) | 0.349 |
| VAT ≥ 82.618 | 7.740 (5.692, 10.601) | < 0.001 | 3.311 (2.217, 4.969) | < 0.001 | 2.578 (1.686, 3.956) | < 0.001 | 2.602 (1.697, 4.004) | < 0.001 |
| SAT ≥ 161.242 | 3.119 (2.341, 4.167) | < 0.001 | 1.296 (0.874, 1.916) | 0.195 | 0.784 (0.503, 1.218) | 0.281 | 0.783 (0.502, 1.216) | 0.279 |
| TAT ≥ 240.849 | 5.667 (4.203, 7.686) | < 0.001 | 2.534 (1.664, 3.869) | < 0.001 | 1.689 (1.043, 2.741) | 0.033 | 1.691 (1.042, 2.747) | 0.033 |
| WC ≥ 85.634 | 5.545 (4.116, 7.512) | < 0.001 | 2.626 (1.724, 4.013) | < 0.001 | - | - | - | - |

The AUC values of 1 for both TAD and SAD in both genders suggest flawless distinction between different samples across all thresholds. In such cases, logistic regression models for binary classification can’t yield meaningful coefficient estimates or odds ratios due to the perfect discrimination. BMI, body mass index; VAT, visceral adipose tissue area; SAT, subcutaneous adipose tissue area; TAT, total abdominal adipose tissue area; WC, waist circumference; OR, odds ratio; 95% CI, 95% confidence interval.

^1^ Adjustment for age and BMI. Weight was adjusted for age and height.

^2^ Adjustment for age and WC.

^3^ Adjustment for age, BMI and WC. Weight was adjusted for age, height and WC.

**Table S25 Association of Unnormalized Body Composition Indicators with Cardio-Cerebrovascular Diseases**

|  | Crude OR (95% CI) | *P* value | Adj. OR (95% CI)^1^ | *P* value | Adj. OR (95% CI)^2^ | *P* value | Adj. OR (95% CI)^3^ | *P* value |
| --- | --- | --- | --- | --- | --- | --- | --- | --- |
| Male |  |  |  |  |  |  |  |  |
| Weight ≥ 68.95 | 1.438 (1.132, 1.830) | 0.003 | 2.104 (1.587, 2.803) | < 0.001 | 0.809 (0.558, 1.170) | 0.261 | 0.958 (0.653, 1.406) | 0.826 |
| VAT ≥ 120.224 | 3.124 (2.418, 4.057) | < 0.001 | 2.945 (2.086, 4.186) | < 0.001 | 2.222 (1.530, 3.241) | < 0.001 | 2.261 (1.553, 3.307) | < 0.001 |
| SAT ≥ 60.309 | 1.459 (1.050, 2.018) | 0.023 | 0.818 (0.531, 1.251) | 0.357 | 0.547 (0.347, 0.853) | 0.008 | 0.573 (0.363, 0.897) | 0.016 |
| TAT ≥ 227.222 | 2.234 (1.753, 2.855) | < 0.001 | 1.996 (1.405, 2.846) | < 0.001 | 1.318 (0.894, 1.945) | 0.164 | 1.368 (0.925, 2.025) | 0.117 |
| WC ≥ 87.027 | 2.592 (2.036, 3.309) | < 0.001 | 2.532 (1.763, 3.652) | < 0.001 | - | - | - | - |
| TAD ≥ 30.897 | 2.326 (1.817, 2.988) | < 0.001 | 2.104 (1.475, 3.014) | < 0.001 | 1.368 (0.912, 2.055) | 0.130 | 1.376 (0.916, 2.070) | 0.125 |
| SAD ≥ 20.953 | 2.701 (2.114, 3.463) | < 0.001 | 2.706 (1.910, 3.856) | < 0.001 | 1.944 (1.313, 2.887) | < 0.001 | 1.952 (1.316, 2.903) | < 0.001 |
| Female |  |  |  |  |  |  |  |  |
| Weight ≥ 58.85 | 2.097 (1.636, 2.693) | < 0.001 | 2.080 (1.527, 2.844) | < 0.001 | 1.228 (0.838, 1.800) | 0.292 | 1.385 (0.932, 2.061) | 0.107 |
| VAT ≥ 65.341 | 4.305 (3.337, 5.576) | < 0.001 | 1.715 (1.212, 2.427) | 0.002 | 1.604 (1.116, 2.308) | 0.011 | 1.584 (1.099, 2.283) | 0.014 |
| SAT ≥ 167.429 | 2.460 (1.904, 3.184) | < 0.001 | 1.145 (0.787, 1.664) | 0.477 | 0.976 (0.648, 1.467) | 0.906 | 0.954 (0.631, 1.438) | 0.822 |
| TAT ≥ 240.849 | 3.514 (2.727, 4.541) | < 0.001 | 1.538 (1.050, 2.253) | 0.027 | 1.353 (0.873, 2.096) | 0.176 | 1.329 (0.856, 2.064) | 0.205 |
| WC ≥ 86.926 | 4.248 (3.246, 5.584) | < 0.001 | 2.373 (1.581, 3.577) | < 0.001 | - | - | - | - |
| TAD ≥ 30.087 | 2.944 (2.290, 3.794) | < 0.001 | 1.336 (0.926, 1.926) | 0.121 | 1.072 (0.685, 1.675) | 0.762 | 1.078 (0.688, 1.685) | 0.743 |
| SAD ≥ 19.270 | 3.751 (2.914, 4.843) | < 0.001 | 2.201 (1.526, 3.182) | < 0.001 | 2.215 (1.438, 3.424) | < 0.001 | 2.207 (1.432, 3.413) | < 0.001 |

BMI, body mass index; VAT, visceral adipose tissue area; SAT, subcutaneous adipose tissue area; TAT, total abdominal adipose tissue area; WC, waist circumference; TAD, transverse diameter; SAD, sagittal diameter; OR, odds ratio; 95% CI, 95% confidence interval.

^1^ Adjustment for age and BMI. Weight was adjusted for age and height.

^2^ Adjustment for age and WC.

^3^ Adjustment for age, BMI and WC. Weight was adjusted for age, height and WC.

**Table S26 Association of Unnormalized Body Composition Indicators with Cancers**

|  | Crude OR (95% CI) | *P* value | Adj. OR (95% CI)^1^ | *P* value | Adj. OR (95% CI)^2^ | *P* value | Adj. OR (95% CI)^3^ | *P* value |
| --- | --- | --- | --- | --- | --- | --- | --- | --- |
| Male |  |  |  |  |  |  |  |  |
| Weight ≥ 84.7 | 1.274 (0.709, 2.369) | 0.429 | 2.354 (1.202, 4.778) | 0.015 | 1.332 (0.630, 2.895) | 0.460 | 1.448 (0.682, 3.160) | 0.342 |
| VAT ≥ 120.195 | 1.879 (1.436, 2.470) | < 0.001 | 1.724 (1.186, 2.518) | 0.005 | 1.305 (0.880, 1.939) | 0.187 | 1.401 (0.939, 2.094) | 0.099 |
| SAT ≥ 59.903 | 1.101 (0.787, 1.533) | 0.570 | 0.878 (0.562, 1.365) | 0.565 | 0.642 (0.404, 1.013) | 0.059 | 0.681 (0.427, 1.081) | 0.105 |
| TAT ≥ 226.134 | 1.435 (1.113, 1.855) | 0.006 | 1.440 (0.988, 2.103) | 0.058 | 1.012 (0.673, 1.521) | 0.954 | 1.090 (0.721, 1.649) | 0.683 |
| WC ≥ 87.148 | 1.792 (1.394, 2.308) | < 0.001 | 1.887 (1.299, 2.755) | < 0.001 | - | - | - | - |
| TAD ≥ 31.420 | 1.700 (1.288, 2.255) | < 0.001 | 1.841 (1.239, 2.755) | 0.003 | 1.341 (0.860, 2.098) | 0.197 | 1.381 (0.883, 2.168) | 0.158 |
| SAD ≥ 21.509 | 2.169 (1.661, 2.846) | < 0.001 | 2.810 (1.935, 4.116) | < 0.001 | 2.460 (1.613, 3.774) | < 0.001 | 2.440 (1.598, 3.751) | < 0.001 |
| Female |  |  |  |  |  |  |  |  |
| Weight ≥ 59.75 | 1.608 (1.246, 2.077) | < 0.001 | 1.653 (1.226, 2.231) | < 0.001 | 1.275 (0.883, 1.841) | 0.195 | 1.461 (1.001, 2.136) | 0.050 |
| VAT ≥ 65.303 | 2.772 (2.173, 3.546) | < 0.001 | 1.297 (0.932, 1.804) | 0.122 | 1.397 (0.989, 1.976) | 0.058 | 1.343 (0.946, 1.905) | 0.099 |
| SAT ≥ 156.256 | 1.811 (1.420, 2.313) | < 0.001 | 1.201 (0.858, 1.681) | 0.285 | 1.303 (0.893, 1.903) | 0.170 | 1.271 (0.869, 1.858) | 0.217 |
| TAT ≥ 235.870 | 2.215 (1.728, 2.845) | < 0.001 | 1.119 (0.781, 1.602) | 0.538 | 1.218 (0.811, 1.829) | 0.342 | 1.165 (0.773, 1.757) | 0.465 |
| WC ≥ 87.647 | 2.619 (1.979, 3.477) | < 0.001 | 1.502 (1.012, 2.237) | 0.044 | - | - | - | - |
| TAD ≥ 28.501 | 1.975 (1.549, 2.523) | < 0.001 | 1.015 (0.726, 1.418) | 0.930 | 1.010 (0.672, 1.517) | 0.960 | 1.030 (0.684, 1.550) | 0.886 |
| SAD ≥ 19.140 | 2.399 (1.874, 3.077) | < 0.001 | 1.459 (1.041, 2.047) | 0.028 | 1.700 (1.144, 2.532) | 0.009 | 1.695 (1.140, 2.526) | 0.009 |

BMI, body mass index; VAT, visceral adipose tissue area; SAT, subcutaneous adipose tissue area; TAT, total abdominal adipose tissue area; WC, waist circumference; TAD, transverse diameter; SAD, sagittal diameter; OR, odds ratio; 95% CI, 95% confidence interval.

^1^ Adjustment for age and BMI. Weight was adjusted for age and height.

^2^ Adjustment for age and WC.

^3^ Adjustment for age, BMI and WC. Weight was adjusted for age, height and WC.

**Table S27 Association of Unnormalized Body Composition Indicators with Abnormal Bone Mass**

|  | Crude OR (95% CI) | *P* value | Adj. OR (95% CI)^1^ | *P* value | Adj. OR (95% CI)^2^ | *P* value | Adj. OR (95% CI)^3^ | *P* value |
| --- | --- | --- | --- | --- | --- | --- | --- | --- |
| Male |  |  |  |  |  |  |  |  |
| Weight ≥ 63.55 | 0.48 (0.329, 0.698) | < 0.001 | 0.924 (0.552, 1.556) | 0.765 | 0.309 (0.153, 0.610) | < 0.001 | 0.359 (0.173, 0.732) | 0.005 |
| VAT ≥ 123.033 | 2.904 (1.974, 4.283) | < 0.001 | 3.146 (1.673, 6.019) | < 0.001 | 1.774 (0.891, 3.557) | 0.104 | 1.825 (0.899, 3.740) | 0.097 |
| SAT ≥ 52.181 | 2.014 (1.063, 4.159) | 0.042 | 2.292 (0.889, 6.321) | 0.096 | 1.121 (0.426, 3.114) | 0.820 | 1.395 (0.518, 3.950) | 0.518 |
| TAT ≥ 243.684 | 1.851 (1.264, 2.709) | 0.002 | 2.019 (1.048, 3.931) | 0.037 | 0.897 (0.430, 1.859) | 0.770 | 0.996 (0.468, 2.114) | 0.992 |
| WC ≥ 86.822 | 2.039 (1.402, 2.981) | < 0.001 | 3.619 (1.865, 7.204) | < 0.001 | - | - | - | - |
| TAD ≥ 31.439 | 2.060 (1.384, 3.058) | < 0.001 | 3.549 (1.787, 7.206) | < 0.001 | 1.787 (0.834, 3.847) | 0.136 | 1.955 (0.892, 4.317) | 0.095 |
| SAD ≥ 20.711 | 1.984 (1.365, 2.892) | < 0.001 | 2.965 (1.564, 5.729) | 0.001 | 1.521 (0.739, 3.154) | 0.256 | 1.593 (0.764, 3.350) | 0.216 |
| Female |  |  |  |  |  |  |  |  |
| Weight ≥ 59.8 | 1.343 (1.011, 1.780) | 0.041 | 1.529 (0.963, 2.437) | 0.073 | 1.072 (0.619, 1.858) | 0.803 | 1.220 (0.694, 2.149) | 0.489 |
| VAT ≥ 74.361 | 6.700 (5.037, 8.969) | < 0.001 | 2.687 (1.618, 4.498) | < 0.001 | 2.358 (1.367, 4.091) | 0.002 | 2.478 (1.431, 4.319) | 0.001 |
| SAT ≥ 146.225 | 1.860 (1.432, 2.419) | < 0.001 | 1.276 (0.773, 2.110) | 0.340 | 0.946 (0.531, 1.681) | 0.850 | 0.963 (0.540, 1.713) | 0.898 |
| TAT ≥ 240.683 | 4.455 (3.385, 5.887) | < 0.001 | 2.323 (1.360, 4.001) | 0.002 | 1.991 (1.067, 3.736) | 0.031 | 2.082 (1.114, 3.919) | 0.022 |
| WC ≥ 83.540 | 3.361 (2.569, 4.412) | < 0.001 | 1.484 (0.875, 2.521) | 0.143 | - | - | - | - |
| TAD ≥ 29.312 | 3.070 (2.346, 4.032) | < 0.001 | 1.395 (0.840, 2.319) | 0.197 | 1.018 (0.540, 1.917) | 0.955 | 0.995 (0.527, 1.874) | 0.988 |
| SAD ≥ 19.149 | 3.635 (2.774, 4.781) | < 0.001 | 1.912 (1.137, 3.230) | 0.015 | 1.582 (0.853, 2.949) | 0.146 | 1.609 (0.867, 2.997) | 0.132 |

BMI, body mass index; VAT, visceral adipose tissue area; SAT, subcutaneous adipose tissue area; TAT, total abdominal adipose tissue area; WC, waist circumference; TAD, transverse diameter; SAD, sagittal diameter; OR, odds ratio; 95% CI, 95% confidence interval.

^1^ Adjustment for age and BMI. Weight was adjusted for age and height.

^2^ Adjustment for age and WC.

^3^ Adjustment for age, BMI and WC. Weight was adjusted for age, height and WC.

**Table S28-1 Association of Height-Normalized Body Composition Indicators with Dyslipidemia Stratified by BMI in Males**

|  | Crude OR (95% CI) | *P* value | Adj. OR (95% CI)^1^ | *P* value | Adj. OR (95% CI)^2^ | *P* value | Adj. OR (95% CI)^3^ | *P* value |
| --- | --- | --- | --- | --- | --- | --- | --- | --- |
| BMI < 24 |  |  |  |  |  |  |  |  |
| VAT/height*^β^* ≥ 34.224 | 3.474 (2.322, 5.278) | < 0.001 | 3.342 (2.126, 5.327) | < 0.001 | 3.065 (1.889, 5.036) | < 0.001 | 3.071 (1.893, 5.044) | < 0.001 |
| SAT/height*^β^* ≥ 31.642 | 1.716 (1.201, 2.462) | 0.003 | 1.439 (0.959, 2.165) | 0.079 | 1.190 (0.771, 1.837) | 0.432 | 1.198 (0.775, 1.853) | 0.415 |
| TAT/height*^β^* ≥ 66.692 | 3.645 (2.458, 5.482) | < 0.001 | 3.675 (2.343, 5.842) | < 0.001 | 3.459 (2.121, 5.713) | < 0.001 | 3.472 (2.130, 5.731) | < 0.001 |
| WC/height*^β^* ≥ 63.194 | 2.869 (1.868, 4.484) | < 0.001 | 2.539 (1.580, 4.140) | < 0.001 | - | - | - | - |
| TAD/height*^β^* ≥ 22.080 | 2.471 (1.614, 3.842) | < 0.001 | 2.110 (1.322, 3.413) | 0.002 | 1.766 (1.057, 2.980) | 0.031 | 1.761 (1.053, 2.972) | 0.032 |
| SAD/height*^β^* ≥ 15.228 | 3.022 (1.945, 4.791) | < 0.001 | 2.693 (1.674, 4.411) | < 0.001 | 2.368 (1.394, 4.084) | 0.002 | 2.366 (1.389, 4.093) | 0.002 |
| BMI ≥ 24 |  |  |  |  |  |  |  |  |
| VAT/height*^β^* ≥ 34.224 | 3.852 (2.435, 6.094) | < 0.001 | 3.126 (1.919, 5.094) | < 0.001 | 2.733 (1.614, 4.627) | < 0.001 | 2.791 (1.646, 4.735) | < 0.001 |
| SAT/height*^β^* ≥ 31.642 | 1.480 (0.892, 2.404) | 0.120 | 1.162 (0.686, 1.930) | 0.568 | 1.013 (0.586, 1.712) | 0.963 | 1.003 (0.581, 1.694) | 0.990 |
| TAT/height*^β^* ≥ 66.692 | 4.923 (2.870, 8.514) | < 0.001 | 3.845 (2.190, 6.802) | < 0.001 | 3.257 (1.761, 6.059) | < 0.001 | 3.421 (1.840, 6.409) | < 0.001 |
| WC/height*^β^* ≥ 63.194 | 4.176 (2.378, 7.361) | < 0.001 | 3.139 (1.740, 5.681) | < 0.001 | - | - | - | - |
| TAD/height*^β^* ≥ 22.080 | 3.188 (1.904, 5.311) | < 0.001 | 2.357 (1.364, 4.053) | 0.002 | 1.818 (0.982, 3.347) | 0.055 | 1.893 (1.019, 3.503) | 0.042 |
| SAD/height*^β^* ≥ 15.228 | 3.035 (1.938, 4.734) | < 0.001 | 2.379 (1.480, 3.812) | < 0.001 | 1.965 (1.153, 3.336) | 0.013 | 2.033 (1.190, 3.468) | 0.009 |

BMI, body mass index; VAT, visceral adipose tissue area; SAT, subcutaneous adipose tissue area; TAT, total abdominal adipose tissue area; WC, waist circumference; TAD, transverse diameter; SAD, sagittal diameter; OR, odds ratio; 95% CI, 95% confidence interval.

^1^ Adjustment for age and BMI.

^2^ Adjustment for age and WC.

^3^ Adjustment for age, BMI and WC.

**Table S28-2 Association of Height-Normalized Body Composition Indicators with Dyslipidemia Stratified by BMI in Females**

|  | Crude OR (95% CI) | *P* value | Adj. OR (95% CI)^1^ | *P* value | Adj. OR (95% CI)^2^ | *P* value | Adj. OR (95% CI)^3^ | *P* value |
| --- | --- | --- | --- | --- | --- | --- | --- | --- |
| BMI < 24 |  |  |  |  |  |  |  |  |
| VAT/height*^β^* ≥ 44.095 | 4.963 (3.481, 7.112) | < 0.001 | 4.747 (3.149, 7.216) | < 0.001 | 5.062 (3.301, 7.840) | < 0.001 | 4.987 (3.248, 7.732) | < 0.001 |
| SAT/height*^β^* ≥ 73.172 | 1.498 (1.021, 2.184) | 0.037 | 1.062 (0.694, 1.613) | 0.780 | 0.932 (0.582, 1.481) | 0.766 | 0.928 (0.581, 1.476) | 0.754 |
| TAT/height*^β^* ≥ 127.606 | 3.052 (1.981, 4.712) | < 0.001 | 2.237 (1.386, 3.616) | < 0.001 | 2.211 (1.298, 3.777) | 0.004 | 2.285 (1.338, 3.918) | 0.003 |
| WC/height*^β^* ≥ 64.509 | 2.787 (1.845, 4.210) | < 0.001 | 2.045 (1.287, 3.251) | 0.002 | - | - | - | - |
| TAD/height*^β^* ≥ 22.852 | 2.457 (1.613, 3.736) | < 0.001 | 1.781 (1.121, 2.822) | 0.014 | 1.685 (0.995, 2.852) | 0.052 | 1.746 (1.029, 2.964) | 0.039 |
| SAD/height*^β^* ≥ 15.341 | 2.806 (1.737, 4.540) | < 0.001 | 2.073 (1.247, 3.446) | 0.005 | 1.933 (1.112, 3.366) | 0.019 | 2.027 (1.162, 3.546) | 0.013 |
| BMI ≥ 24 |  |  |  |  |  |  |  |  |
| VAT/height*^β^* ≥ 44.095 | 3.378 (1.820, 6.452 | < 0.001 | 2.062 (1.045, 4.140) | 0.039 | 1.974 (0.982, 4.037) | 0.058 | 1.897 (0.937, 3.903) | 0.077 |
| SAT/height*^β^* ≥ 73.172 | 2.243 (1.245, 4.086) | 0.008 | 1.529 (0.812, 2.892) | 0.189 | 1.406 (0.731, 2.712) | 0.307 | 1.375 (0.709, 2.679) | 0.346 |
| TAT/height*^β^* ≥ 127.606 | 2.926 (1.627, 5.361) | < 0.001 | 1.761 (0.927, 3.381) | 0.085 | 1.581 (0.794, 3.170) | 0.193 | 1.583 (0.789, 3.198) | 0.197 |
| WC/height*^β^* ≥ 64.509 | 4.418 (2.151, 9.664) | < 0.001 | 2.572 (1.177, 5.901) | 0.021 | - | - | - | - |
| TAD/height*^β^* ≥ 22.852 | 1.984 (1.043, 3.810) | 0.037 | 1.063 (0.522, 2.162) | 0.865 | 0.771 (0.350, 1.685) | 0.515 | 0.820 (0.371, 1.806) | 0.622 |
| SAD/height*^β^* ≥ 15.341 | 4.476 (2.647, 7.710) | < 0.001 | 3.058 (1.724, 5.493) | < 0.001 | 3.147 (1.678, 5.993) | < 0.001 | 3.203 (1.691, 6.174) | < 0.001 |

BMI, body mass index; VAT, visceral adipose tissue area; SAT, subcutaneous adipose tissue area; TAT, total abdominal adipose tissue area; WC, waist circumference; TAD, transverse diameter; SAD, sagittal diameter; OR, odds ratio; 95% CI, 95% confidence interval.

^1^ Adjustment for age and BMI.

^2^ Adjustment for age and WC.

^3^ Adjustment for age, BMI and WC.

**Table S29-1 Association of Height-Normalized Body Composition Indicators with Type 2 Diabetes Stratified by BMI in Males**

|  | Crude OR (95% CI) | *P* value | Adj. OR (95% CI)^1^ | *P* value | Adj. OR (95% CI)^2^ | *P* value | Adj. OR (95% CI)^3^ | *P* value |
| --- | --- | --- | --- | --- | --- | --- | --- | --- |
| BMI < 24 |  |  |  |  |  |  |  |  |
| VAT/height*^β^* ≥ 34.824 | 4.940 (3.223, 7.706) | < 0.001 | 3.727 (2.295, 6.145) | < 0.001 | 2.818 (1.666, 4.827) | < 0.001 | 2.815 (1.663, 4.825) | < 0.001 |
| SAT/height*^β^* ≥ 23.272 | 1.511 (1.043, 2.199) | 0.030 | 1.023 (0.629, 1.662) | 0.927 | 0.579 (0.340, 0.974) | 0.042 | 0.629 (0.365, 1.074) | 0.092 |
| TAT/height*^β^* ≥ 66.728 | 3.615 (2.395, 5.526) | < 0.001 | 2.860 (1.767, 4.685) | < 0.001 | 2.003 (1.182, 3.420) | 0.010 | 2.034 (1.198, 3.480) | 0.009 |
| WC/height*^β^* ≥ 63.088 | 3.877 (2.536, 6.019) | < 0.001 | 2.846 (1.730, 4.741) | < 0.001 | - | - | - | - |
| BMI ≥ 24 |  |  |  |  |  |  |  |  |
| VAT/height*^β^* ≥ 34.824 | 5.354 (3.117, 9.425) | < 0.001 | 4.039 (2.255, 7.385) | < 0.001 | 2.656 (1.402, 5.101) | 0.003 | 2.632 (1.388, 5.057) | 0.003 |
| SAT/height*^β^* ≥ 23.272 | 1.511 (0.489, 4.428) | 0.453 | 1.162 (0.354, 3.642) | 0.797 | 0.722 (0.204, 2.413) | 0.6 | 0.720 (0.202, 2.411) | 0.598 |
| TAT/height*^β^* ≥ 66.728 | 4.595 (2.520, 8.636) | < 0.001 | 3.196 (1.680, 6.231) | < 0.001 | 1.814 (0.888, 3.760) | 0.104 | 1.781 (0.869, 3.702) | 0.117 |
| WC/height*^β^* ≥ 63.088 | 6.950 (3.386, 15.433) | < 0.001 | 4.833 (2.260, 11.084) | < 0.001 | - | - | - | - |

The AUC values of 1 for both TAD (TAD/height*^β^*) and SAD (SAD/height*^β^*) in both genders suggest flawless distinction between different samples across all thresholds. In such cases, logistic regression models for binary classification can't yield meaningful coefficient estimates or odds ratios due to the perfect discrimination. BMI, body mass index; VAT, visceral adipose tissue area; SAT, subcutaneous adipose tissue area; TAT, total abdominal adipose tissue area; WC, waist circumference; OR, odds ratio; 95% CI, 95% confidence interval.

^1^ Adjustment for age and BMI.

^2^ Adjustment for age and WC.

^3^ Adjustment for age, BMI and WC.

**Table S29-2 Association of Height-Normalized Body Composition Indicators with Type 2 Diabetes Stratified by BMI in Females**

|  | Crude OR (95% CI) | *P* value | Adj. OR (95% CI)^1^ | *P* value | Adj. OR (95% CI)^2^ | *P* value | Adj. OR (95% CI)^3^ | *P* value |
| --- | --- | --- | --- | --- | --- | --- | --- | --- |
| BMI < 24 |  |  |  |  |  |  |  |  |
| VAT/height*^β^* ≥ 47.488 | 6.839 (4.547, 10.351) | < 0.001 | 4.583 (2.826, 7.498) | < 0.001 | 3.331 (2.016, 5.533) | < 0.001 | 3.584 (2.149, 6.017) | < 0.001 |
| SAT/height*^β^* ≥ 72.682 | 1.970 (1.299, 2.966) | 0.001 | 1.420 (0.861, 2.331) | 0.166 | 0.773 (0.442, 1.339) | 0.362 | 0.799 (0.454, 1.393) | 0.433 |
| TAT/height*^β^* ≥ 113.968 | 4.545 (3.058, 6.775) | < 0.001 | 3.387 (2.085, 5.536) | < 0.001 | 2.244 (1.265, 4.004) | 0.006 | 2.283 (1.281, 4.090) | 0.005 |
| WC/height*^β^* ≥ 64.652 | 5.683 (3.663, 8.859) | < 0.001 | 4.054 (2.390, 6.927) | < 0.001 | - | - | - | - |
| BMI ≥ 24 |  |  |  |  |  |  |  |  |
| VAT/height*^β^* ≥ 47.488 | 4.262 (2.226, 8.581) | < 0.001 | 1.886 (0.887, 4.116) | 0.103 | 1.619 (0.734, 3.641) | 0.236 | 1.539 (0.694, 3.473) | 0.291 |
| SAT/height*^β^* ≥ 72.682 | 1.947 (1.056, 3.649) | 0.034 | 1.231 (0.600, 2.531) | 0.570 | 0.929 (0.438, 1.968) | 0.847 | 0.935 (0.438, 1.998) | 0.862 |
| TAT/height*^β^* ≥ 113.968 | 3.099 (1.286, 8.269) | 0.016 | 1.041 (0.363, 3.162) | 0.941 | 0.728 (0.242, 2.299) | 0.576 | 0.709 (0.233, 2.260) | 0.549 |
| WC/height*^β^* ≥ 64.652 | 4.168 (1.940, 9.764) | < 0.001 | 1.821 (0.727, 4.798) | 0.209 | - | - | - | - |

The AUC values of 1 for both TAD (TAD/height*^β^*) and SAD (SAD/height*^β^*) in both genders suggest flawless distinction between different samples across all thresholds. In such cases, logistic regression models for binary classification can't yield meaningful coefficient estimates or odds ratios due to the perfect discrimination. BMI, body mass index; VAT, visceral adipose tissue area; SAT, subcutaneous adipose tissue area; TAT, total abdominal adipose tissue area; WC, waist circumference; OR, odds ratio; 95% CI, 95% confidence interval.

^1^ Adjustment for age and BMI.

^2^ Adjustment for age and WC.

^3^ Adjustment for age, BMI and WC.

**Table S30-1 Association of Height-Normalized Body Composition Indicators with Cardio-Cerebrovascular Diseases Stratified by BMI in Males**

|  | Crude OR (95% CI) | *P* value | Adj. OR (95% CI)^1^ | *P* value | Adj. OR (95% CI)^2^ | *P* value | Adj. OR (95% CI)^3^ | *P* value |
| --- | --- | --- | --- | --- | --- | --- | --- | --- |
| BMI < 24 |  |  |  |  |  |  |  |  |
| VAT/height*^β^* ≥ 36.959 | 3.508 (2.283, 5.557) | < 0.001 | 3.567 (2.184, 5.984) | < 0.001 | 2.699 (1.596, 4.671) | < 0.001 | 2.555 (1.505, 4.433) | < 0.001 |
| SAT/height*^β^* ≥ 45.681 | 1.001 (0.416, 2.561) | 0.997 | 1.091 (0.413, 3.056) | 0.863 | 0.750 (0.281, 2.123) | 0.573 | 0.632 (0.234, 1.815) | 0.375 |
| TAT/height*^β^* ≥ 68.798 | 2.228 (1.511, 3.343) | < 0.001 | 2.626 (1.649, 4.253) | < 0.001 | 1.807 (1.091, 3.031) | 0.023 | 1.777 (1.067, 2.996) | 0.029 |
| WC/height*^β^* ≥ 63.842 | 2.933 (1.876, 4.733) | < 0.001 | 3.327 (1.976, 5.758) | < 0.001 | - | - | - | - |
| TAD/height*^β^* ≥ 22.645 | 4.170 (2.297, 8.230) | < 0.001 | 4.027 (2.108, 8.299) | < 0.001 | 3.138 (1.565, 6.739) | 0.002 | 2.433 (1.206, 5.233) | 0.017 |
| SAD/height*^β^* ≥ 15.319 | 3.283 (2.156, 5.138) | < 0.001 | 4.137 (2.527, 6.9546) | < 0.001 | 3.263 (1.898, 5.737) | < 0.001 | 2.943 (1.702, 5.198) | < 0.001 |
| BMI ≥ 24 |  |  |  |  |  |  |  |  |
| VAT/height*^β^* ≥ 36.959 | 2.984 (1.955, 4.555) | < 0.001 | 2.016 (1.249, 3.245) | 0.004 | 1.526 (0.923, 2.564) | 0.112 | 1.550 (0.916, 2.606) | 0.100 |
| SAT/height*^β^* ≥ 45.681 | 1.350 (0.911, 2.019) | 0.139 | 1.172 (0.726, 1.910) | 0.52 | 1.053 (0.648, 1.722) | 0.835 | 1.015 (0.620, 1.672) | 0.954 |
| TAT/height*^β^* ≥ 68.798 | 3.282 (1.986, 5.426) | < 0.001 | 2.394 (1.354, 4.230) | 0.003 | 1.737 (0.929, 3.226) | 0.082 | 1.769 (0.946, 3.291) | 0.072 |
| WC/height*^β^* ≥ 63.842 | 4.776 (2.774, 8.331) | < 0.001 | 3.161 (1.714, 5.879) | < 0.001 | - | - | - | - |
| TAD/height*^β^* ≥ 22.645 | 2.646 (1.710, 4.086) | < 0.001 | 1.989 (1.197, 3.303) | 0.008 | 1.390 (0.779, 2.467) | 0.261 | 1.412 (0.791, 2.510) | 0.240 |
| SAD/height*^β^* ≥ 15.319 | 3.537 (2.232, 5.615) | < 0.001 | 2.562 (1.520, 4.320) | < 0.001 | 1.892 (1.058, 3.371) | 0.031 | 1.947 (1.086, 3.481) | 0.025 |

BMI, body mass index; VAT, visceral adipose tissue area; SAT, subcutaneous adipose tissue area; TAT, total abdominal adipose tissue area; WC, waist circumference; TAD, transverse diameter; SAD, sagittal diameter; OR, odds ratio; 95% CI, 95% confidence interval.

^1^ Adjustment for age and BMI.

^2^ Adjustment for age and WC.

^3^ Adjustment for age, BMI and WC.

**Table S30-2 Association of Height-Normalized Body Composition Indicators with Cardio-Cerebrovascular Diseases Stratified by BMI in Females**

|  | Crude OR (95% CI) | *P* value | Adj. OR (95% CI)^1^ | *P* value | Adj. OR (95% CI)^2^ | *P* value | Adj. OR (95% CI)^3^ | *P* value |
| --- | --- | --- | --- | --- | --- | --- | --- | --- |
| BMI < 24 |  |  |  |  |  |  |  |  |
| VAT/height*^β^* ≥ 37.717 | 3.956 (2.888, 5.441) | < 0.001 | 2.532 (1.703, 3.783) | < 0.001 | 2.132 (1.414, 3.226) | < 0.001 | 2.310 (1.520, 3.526) | < 0.001 |
| SAT/height*^β^* ≥ 79.154 | 1.475 (0.946, 2.282) | 0.083 | 1.139 (0.672, 1.921) | 0.627 | 0.871 (0.495, 1.525) | 0.630 | 0.852 (0.481, 1.497) | 0.578 |
| TAT/height*^β^* ≥ 110.633 | 2.517 (1.809, 3.506) | < 0.001 | 1.846 (1.199, 2.850) | 0.005 | 1.425 (0.869, 2.338) | 0.160 | 1.515 (0.920, 2.500) | 0.103 |
| WC/height*^β^* ≥ 65.280 | 3.614 (2.362, 5.582) | < 0.001 | 2.387 (1.431, 4.017) | < 0.001 | - | - | - | - |
| TAD/height*^β^* ≥ 22.688 | 2.466 (1.693, 3.598) | < 0.001 | 1.760 (1.108, 2.802) | 0.017 | 1.427 (0.827, 2.465) | 0.201 | 1.360 (0.785, 2.358) | 0.272 |
| SAD/height*^β^* ≥ 14.649 | 3.122 (2.217, 4.409) | < 0.001 | 2.994 (1.926, 4.688) | < 0.001 | 2.777 (1.673, 4.648) | < 0.001 | 2.885 (1.730, 4.848) | < 0.001 |
| BMI ≥ 24 |  |  |  |  |  |  |  |  |
| VAT/height*^β^* ≥ 37.717 | 1.845 (0.924, 3.697) | 0.081 | 0.598 (0.241, 1.443) | 0.258 | 0.551 (0.217, 1.359) | 0.201 | 0.579 (0.225, 1.458) | 0.250 |
| SAT/height*^β^* ≥ 79.154 | 1.763 (1.077, 2.887) | 0.024 | 1.091 (0.585, 2.017) | 0.783 | 1.144 (0.585, 2.225) | 0.692 | 1.127 (0.574, 2.200) | 0.727 |
| TAT/height*^β^* ≥ 110.633 | 1.402 (0.670, 2.895) | 0.362 | 0.373 (0.136, 0.976) | 0.049 | 0.286 (0.099, 0.793) | 0.018 | 0.313 (0.106, 0.897) | 0.033 |
| WC/height*^β^* ≥ 65.280 | 2.470 (1.373, 4.492) | 0.003 | 0.953 (0.436, 2.046) | 0.903 | - | - | - | - |
| TAD/height*^β^* ≥ 22.688 | 1.262 (0.675, 2.333) | 0.459 | 0.424 (0.179, 0.959) | 0.044 | 0.289 (0.110, 0.729) | 0.010 | 0.332 (0.124, 0.848) | 0.024 |
| SAD/height*^β^* ≥ 14.649 | 3.708 (2.001, 7.070) | < 0.001 | 1.751 (0.819, 3.768) | 0.149 | 1.849 (0.795, 4.348) | 0.154 | 2.118 (0.893, 5.120) | 0.091 |

BMI, body mass index; VAT, visceral adipose tissue area; SAT, subcutaneous adipose tissue area; TAT, total abdominal adipose tissue area; WC, waist circumference; TAD, transverse diameter; SAD, sagittal diameter; OR, odds ratio; 95% CI, 95% confidence interval.

^1^ Adjustment for age and BMI.

^2^ Adjustment for age and WC.

^3^ Adjustment for age, BMI and WC.

**Table 31-1 Association of Height-Normalized Body Composition Indicators with Cancers Stratified by BMI in Males**

|  | Crude OR (95% CI) | *P* value | Adj. OR (95% CI)^1^ | *P* value | Adj. OR (95% CI)^2^ | *P* value | Adj. OR (95% CI)^3^ | *P* value |
| --- | --- | --- | --- | --- | --- | --- | --- | --- |
| BMI < 24 |  |  |  |  |  |  |  |  |
| VAT/height*^β^* ≥ 40.088 | 2.563 (1.508, 4.560) | < 0.001 | 1.970 (1.087, 3.717) | 0.030 | 1.508 (0.810, 2.908) | 0.206 | 1.506 (0.804, 2.916) | 0.211 |
| SAT/height*^β^* ≥ 45.633 | 1.096 (0.455, 2.804) | 0.841 | 1.298 (0.485, 3.691) | 0.610 | 0.994 (0.368, 2.845) | 0.991 | 0.973 (0.356, 2.814) | 0.958 |
| TAT/height*^β^* ≥ 68.293 | 1.571 (1.046, 2.392) | 0.0320 | 1.528 (0.936, 2.529) | 0.094 | 1.114 (0.654, 1.914) | 0.692 | 1.110 (0.649, 1.915) | 0.705 |
| WC/height*^β^* ≥ 63.790 | 2.762 (1.765, 4.449) | < 0.001 | 2.708 (1.589, 4.737) | < 0.001 | - | - | - | - |
| TAD/height*^β^* ≥ 22.645 | 3.787 (2.066, 7.527) | < 0.001 | 3.153 (1.608, 6.648) | 0.001 | 2.617 (1.255, 5.811) | 0.013 | 2.215 (1.052, 4.947) | 0.043 |
| SAD/height*^β^* ≥ 15.233 | 3.344 (2.208, 5.191) | < 0.001 | 3.664 (2.247, 6.130) | < 0.001 | 3.676 (2.100, 6.577) | < 0.001 | 3.272 (1.853, 5.896) | < 0.001 |
| BMI ≥ 24 |  |  |  |  |  |  |  |  |
| VAT/height*^β^* ≥ 40.088 | 2.279 (1.470, 3.547) | < 0.001 | 1.482 (0.879, 2.490) | 0.138 | 1.259 (0.713, 2.208) | 0.423 | 1.254 (0.710, 2.202) | 0.431 |
| SAT/height*^β^* ≥ 45.633 | 1.191 (0.775, 1.843) | 0.427 | 1.408 (0.827, 2.427) | 0.211 | 1.311 (0.766, 2.268) | 0.327 | 1.277 (0.742, 2.224) | 0.381 |
| TAT/height*^β^* ≥ 68.293 | 2.380 (1.389, 4.113) | 0.002 | 1.927 (1.033, 3.613) | 0.040 | 1.628 (0.824, 3.205) | 0.159 | 1.637 (0.829, 3.224) | 0.153 |
| WC/height*^β^* ≥ 63.790 | 2.945 (1.682, 5.238) | < 0.001 | 2.113 (1.102, 4.089) | 0.025 | - | - | - | - |
| TAD/height*^β^* ≥ 22.645 | 1.698 (1.074, 2.685) | 0.023 | 1.166 (0.669, 2.023) | 0.585 | 0.895 (0.478, 1.658) | 0.726 | 0.889 (0.474, 1.650) | 0.711 |
| SAD/height*^β^* ≥ 15.233 | 2.368 (1.447, 3.897) | < 0.001 | 1.915 (1.078, 3.412) | 0.027 | 1.610 (0.848, 3.051) | 0.144 | 1.636 (0.860, 3.107) | 0.132 |

BMI, body mass index; VAT, visceral adipose tissue area; SAT, subcutaneous adipose tissue area; TAT, total abdominal adipose tissue area; WC, waist circumference; TAD, transverse diameter; SAD, sagittal diameter; OR, odds ratio; 95% CI, 95% confidence interval.

^1^ Adjustment for age and BMI.

^2^ Adjustment for age and WC.

^3^ Adjustment for age, BMI and WC.

**Table S31-2 Association of Height-Normalized Body Composition Indicators with Cancers Stratified by BMI in Females**

|  | Crude OR (95% CI) | *P* value | Adj. OR (95% CI)^1^ | *P* value | Adj. OR (95% CI)^2^ | *P* value | Adj. OR (95% CI)^3^ | *P* value |
| --- | --- | --- | --- | --- | --- | --- | --- | --- |
| BMI < 24 |  |  |  |  |  |  |  |  |
| VAT/height*^β^* ≥ 37.894 | 2.687 (1.993, 3.633) | < 0.001 | 1.638 (1.126, 2.387) | 0.010 | 1.509 (1.019, 2.238) | 0.040 | 1.582 (1.065, 2.354) | 0.023 |
| SAT/height*^β^* ≥ 70.819 | 1.425 (1.028, 1.972) | 0.033 | 1.316 (0.875, 1.981) | 0.187 | 1.155 (0.741, 1.801) | 0.525 | 1.213 (0.775, 1.900) | 0.398 |
| TAT/height*^β^* ≥ 110.015 | 1.986 (1.445, 2.731) | < 0.001 | 1.459 (0.970, 2.195) | 0.070 | 1.327 (0.837, 2.109) | 0.229 | 1.374 (0.864, 2.186) | 0.180 |
| WC/height*^β^* ≥ 62.301 | 1.995 (1.477, 2.697) | < 0.001 | 1.791 (1.205, 2.669) | 0.004 | - | - | - | - |
| TAD/height*^β^* ≥ 21.703 | 1.679 (1.257, 2.244) | < 0.001 | 1.377 (0.943, 2.014) | 0.098 | 1.282 (0.802, 2.054) | 0.299 | 1.295 (0.808, 2.080) | 0.283 |
| SAD/height*^β^* ≥ 14.684 | 2.207 (1.571, 3.108) | < 0.001 | 1.823 (1.202, 2.775) | 0.005 | 1.839 (1.152, 2.947) | 0.011 | 1.821 (1.137, 2.926) | 0.013 |
| BMI ≥ 24 |  |  |  |  |  |  |  |  |
| VAT/height*^β^* ≥ 37.894 | 2.278 (1.095, 4.889) | 0.030 | 1.122 (0.477, 2.660) | 0.792 | 1.303 (0.559, 3.079) | 0.540 | 1.279 (0.526, 3.188) | 0.590 |
| SAT/height*^β^* ≥ 70.819 | 1.581 (0.839, 2.994) | 0.156 | 1.213 (0.598, 2.449) | 0.590 | 1.400 (0.672, 2.924) | 0.368 | 1.498 (0.703, 3.230) | 0.297 |
| TAT/height*^β^* ≥ 110.015 | 1.642 (0.730, 3.747) | 0.230 | 0.799 (0.303, 2.081) | 0.644 | 0.802 (0.300, 2.125) | 0.656 | 1.011 (0.359, 2.910) | 0.983 |
| WC/height*^β^* ≥ 62.301 | 1.538 (0.601, 3.990) | 0.366 | 1.047 (0.353, 3.095) | 0.933 | - | - | - | - |
| TAD/height*^β^* ≥ 21.703 | 1.365 (0.456, 4.091) | 0.57 | 0.614 (0.162, 2.273) | 0.464 | 0.467 (0.123, 1.731) | 0.254 | 0.858 (0.202, 3.726) | 0.834 |
| SAD/height*^β^* ≥ 14.684 | 3.134 (1.689, 5.986) | < 0.001 | 2.094 (1.054, 4.228) | 0.036 | 2.843 (1.349, 6.141) | 0.007 | 3.241 (1.485, 7.336) | 0.004 |

BMI, body mass index; VAT, visceral adipose tissue area; SAT, subcutaneous adipose tissue area; TAT, total abdominal adipose tissue area; WC, waist circumference; TAD, transverse diameter; SAD, sagittal diameter; OR, odds ratio; 95% CI, 95% confidence interval.

^1^ Adjustment for age and BMI.

^2^ Adjustment for age and WC.

^3^ Adjustment for age, BMI and WC.

**Table S32-1 Association of Height-Normalized Body Composition Indicators with Abnormal Bone Mass Stratified by BMI in Males**

|  | Crude OR (95% CI) | *P* value | Adj. OR (95% CI)^1^ | *P* value | Adj. OR (95% CI)^2^ | *P* value | Adj. OR (95% CI)^3^ | *P* value |
| --- | --- | --- | --- | --- | --- | --- | --- | --- |
| BMI < 24 |  |  |  |  |  |  |  |  |
| VAT/height*^β^* ≥ 32.740 | 4.390 (2.697, 7.203) | < 0.001 | 2.895 (1.451, 5.855) | 0.003 | 1.829 (0.845, 3.973) | 0.125 | 1.688 (0.764, 3.732) | 0.194 |
| SAT/height*^β^* ≥ 22.830 | 1.701 (1.053, 2.799) | 0.033 | 1.249 (0.589, 2.692) | 0.565 | 0.538 (0.238, 1.207) | 0.132 | 0.634 (0.274, 1.469) | 0.285 |
| TAT/height*^β^* ≥ 57.282 | 2.921 (1.851, 4.650) | < 0.001 | 2.382 (1.184, 4.890) | 0.016 | 1.231 (0.566, 2.691) | 0.601 | 1.293 (0.581, 2.896) | 0.529 |
| WC/height*^β^* ≥ 62.677 | 4.336 (2.635, 7.192) | < 0.001 | 4.313 (2.052, 9.341) | < 0.001 | - | - | - | - |
| TAD/height*^β^* ≥ 21.894 | 3.516 (2.151, 5.776) | < 0.001 | 4.701 (2.197, 10.408) | < 0.001 | 2.799 (1.197, 6.671) | 0.018 | 2.885 (1.209, 7.024) | 0.018 |
| SAD/height*^β^* ≥ 14.470 | 3.406 (2.149, 5.444) | < 0.001 | 2.686 (1.339, 5.508) | 0.006 | 1.464 (0.669, 3.219) | 0.340 | 1.509 (0.677, 3.385) | 0.314 |
| BMI ≥ 24 |  |  |  |  |  |  |  |  |
| VAT/height*^β^* ≥ 32.740 | 5.519 (1.872, 23.657) | 0.006 | 3.265 (0.844, 16.827) | 0.112 | 1.872 (0.439, 10.217) | 0.424 | 1.826 (0.429, 9.926) | 0.441 |
| SAT/height*^β^* ≥ 22.830 | - | - | - | - | - | - | - | - |
| TAT/height*^β^* ≥ 57.282 | - | - | - | - | - | - | - | - |
| WC/height*^β^* ≥ 62.677 | - | - | - | - | - | - | - | - |
| TAD/height*^β^* ≥ 21.894 | - | - | - | - | - | - | - | - |
| SAD/height*^β^* ≥ 14.470 | - | - | - | - | - | - | - | - |

The case group with abnormal bone mass in females with BMI ≥ 24 included 199 individuals of SAT/height*^β^*, 220 of TAT/height*^β^*, 208 of WC/height*^β^*, 217 of TAD/height*^β^*, and 188 of SAD/height*^β^*. Similarly, the control group comprised 35 individuals of SAT/height*^β^*, 14 of TAT/height*^β^*, 26 of WC/height*^β^*, 17 of TAD/height*^β^*, and 46 of SAD/height*^β^*. Due to the small sample size of these indices, logistic regression cannot be performed.

BMI, body mass index; VAT, visceral adipose tissue area; SAT, subcutaneous adipose tissue area; TAT, total abdominal adipose tissue area; WC, waist circumference; TAD, transverse diameter; SAD, sagittal diameter; OR, odds ratio; 95% CI, 95% confidence interval.

^1^ Adjustment for age and BMI.

^2^ Adjustment for age and WC.

^3^ Adjustment for age, BMI and WC.

**Table S32-2 Association of Height-Normalized Body Composition Indicators with Abnormal Bone Mass Stratified by BMI in Females**

|  | Crude OR (95% CI) | *P* value | Adj. OR (95% CI)^1^ | *P* value | Adj. OR (95% CI)^2^ | *P* value | Adj. OR (95% CI)^3^ | *P* value |
| --- | --- | --- | --- | --- | --- | --- | --- | --- |
| BMI < 24 |  |  |  |  |  |  |  |  |
| VAT/height*^β^* ≥ 44.030 | 8.781 (6.209, 12.529) | < 0.001 | 4.129 (2.298, 7.522) | < 0.001 | 2.859 (1.575, 5.227) | < 0.001 | 3.444 (1.865, 6.433) | < 0.001 |
| SAT/height*^β^* ≥ 69.707 | 1.957 (1.404, 2.726) | < 0.001 | 1.076 (0.604, 1.909) | 0.803 | 0.635 (0.332, 1.196) | 0.163 | 0.663 (0.344, 1.259) | 0.213 |
| TAT/height*^β^* ≥ 108.829 | 5.178 (3.730, 7.226) | < 0.001 | 3.320 (1.825, 6.123) | < 0.001 | 2.302 (1.169, 4.572) | 0.016 | 2.539 (1.282, 5.082) | 0.008 |
| WC/height*^β^* ≥ 63.899 | 5.107 (3.611, 7.266) | < 0.001 | 2.494 (1.352, 4.642) | 0.004 | - | - | - | - |
| TAD/height*^β^* ≥ 22.280 | 4.109 (2.961, 5.724) | < 0.001 | 2.188 (1.212, 3.972) | 0.010 | 1.383 (0.693, 2.762) | 0.357 | 1.436 (0.717, 2.876) | 0.306 |
| SAD/height*^β^* ≥ 14.856 | 4.359 (3.038, 6.291) | < 0.001 | 2.041 (1.102, 3.802) | 0.024 | 1.411 (0.702, 2.838) | 0.333 | 1.324 (0.654, 2.680) | 0.434 |
| BMI ≥ 24 |  |  |  |  |  |  |  |  |
| VAT/height*^β^* ≥ 44.030 | 7.609 (3.089, 22.978) | < 0.001 | 1.327 (0.364, 5.265) | 0.673 | 1.296 (0.327, 5.708) | 0.719 | 1.427 (0.347, 6.749) | 0.634 |
| SAT/height*^β^* ≥ 69.707 | 1.675 (0.809, 3.594) | 0.172 | 0.896 (0.268, 2.961) | 0.856 | 0.811 (0.220, 3.002) | 0.752 | 0.884 (0.236, 3.322) | 0.854 |
| TAT/height*^β^* ≥ 108.829 | 6.000 (1.588, 39.137) | 0.021 | 1.402 (0.182, 1.389) | 0.755 | 1.009 (0.125, 11.022) | 0.994 | 1.650 (0.159, 26.390) | 0.700 |
| WC/height*^β^* ≥ 63.899 | 3.464 (1.411, 9.788) | 0.011 | 0.923 (0.230, 3.669) | 0.908 | - | - | - | - |
| TAD/height*^β^* ≥ 22.280 | 2.335 (0835, 7.543) | 0.123 | 0.630 (0.105, 3.443) | 0.602 | 0.420 (0.061, 2.810) | 0.373 | 0.535 (0.007, 4.072) | 0.548 |
| SAD/height*^β^* ≥ 14.856 | 5.881 (2.728, 14.195) | < 0.001 | 1.604 (0.516, 5.250) | 0.420 | 1.812 (0.495, 7.166) | 0.379 | 1.927 (0.515, 7.872) | 0.341 |

BMI, body mass index; VAT, visceral adipose tissue area; SAT, subcutaneous adipose tissue area; TAT, total abdominal adipose tissue area; WC, waist circumference; TAD, transverse diameter; SAD, sagittal diameter; OR, odds ratio; 95% CI, 95% confidence interval.

^1^ Adjustment for age and BMI.

^2^ Adjustment for age and WC.

^3^ Adjustment for age, BMI and WC.
